# Correction: Temporal trends and socioeconomic differences in acute respiratory infection hospitalisations in children: an intercountry comparison of birth cohort studies in Western Australia, England and Scotland

**DOI:** 10.1136/bmjopen-2018-028710corr1

**Published:** 2020-01-26

Moore HC, de Klerk N, Blyth CC, *et al*. Temporal trends and socioeconomic differences in acute respiratory infection hospitalisations in children: an intercountry comparison of birth cohort studies in Western Australia, England and Scotland. *BMJ Open* 2019;9:e028710. doi: 10.1136/bmjopen-2018-028710

This article was previously published with incomplete information in the funding.

The updated funding is below:

This work was supported by a National Health and Medical Research Council Project Grant (1045668), a University of Western Australia Research Collaboration Award (to HCM) and a Wesfarmers Centre of Vaccines and Infectious Diseases Seed Grant (to HCM, CCB, and PH). CCB and HCM are supported by National Health and Medical Research Council Fellowships (1 034 254 to HCM and 1 111 596 to CCB). PH was funded by a National Institute for Health Research postdoctoral fellowship, reference number PDF-2013-06-004. AZ’s PhD studentship is funded by awards to establish the Farr Institute of Health Informatics Research, London, from the Medical Research Council, Arthritis Research UK, British Heart Foundation, Cancer Research UK, Chief Scientist Office, Economic and Social Research Council, Engineering and Physical Sciences Research Council, National Institute for Health Research, National Institute for Social Care and Health Research, and Wellcome Trust (grant MR/K006584/1). AZ is also supported by the Administrative Data Research Centre for England (funded by the Economic and Social Research Council). MV’s PhD studentship is funded by the UBEL DTP of the Economic and Social Research Council. MV was also supported by the Policy Research Unit in the Health of Children, Young People and Families (reference 109/00017), which is funded by the Department of Health Policy Research Programme at UCL. This is an independent report commissioned and funded by the Department of Health. Research at UCL Great Ormond Street Institute of Child Health is supported by the NIHR Great Ormond Street Hospital Biomedical Research Centre.

