# Supplementary material for: Temporal trends and socioeconomic differences in acute respiratory infection hospitalisations in children: an intercountry comparison of birth cohort studies in Western Australia, England and Scotland
Source: BMJ Open. 2019 May 19;9(5):e028710. doi: 10.1136/bmjopen-2018-028710 (PMC6530403; doi:10.1136/bmjopen-2018-028710)
Supplement: Supplementary data [file bmjopen-2018-028710supp001.pdf]

Supplementary Table 1: Cohort characteristics by jurisdiction

Characteristic		Western Australia		England		Scotland	
		n	(%)	n	(%)	n	(%)
Sex[a]	Male	173,081	(51.2)	3,044,931	(51.3)	359,159	(51.3)
	Female	164,828	(48.8)	2,894,078	(48.7)	340,430	(48.7)
Year of birth	2000	22,551	(6.7)	-		51,479	(7.4)
	2001	22,461	(6.7)	-		50,295	(7.2)
	2002	22,412	(6.7)	-		49,425	(7.1)
	2003	22,345	(6.6)	535,724	(9.0)	50,450	(7.2)
	2004	23,361	(6.9)	551,939	(9.3)	52,417	(7.5)
	2005	24,776	(7.3)	560,894	(9.4)	52,415	(7.5)
	2006	26,627	(7.9)	579,220	(9.8)	53,830	(7.7)
	2007	26,943	(8.0)	586,526	(9.9)	55,765	(8.0)
	2008	28,216	(8.4)	614,471	(10.4)	57,481	(8.2)
	2009	28,588	(8.5)	611,427	(10.3)	57,007	(8.2)
	2010	28,565	(8.5)	629,778	(10.7)	56,586	(8.1)
	2011	29,535	(8.7)	632,306	(10.7)	56,510	(8.1)
	2012	31,529	(9.3)	636,724	(10.7)	55,929	(8.0)
Deprivation[b]	<10% (most deprived)	40,936	(12.1)	885,891	(14.9)	85,987	(12.3)
	10-49%	122,309	(36.2)	2,635,562	(44.4)	295,372	(42.2)
	50-89%	131,232	(38.8)	1,957,789	(33.0)	252,407	(36.1)
	≥90% (least deprived)	43,432	(12.9)	459,767	(7.6)	65,823	(9.4)
**TOTAL**		**337,909**	**(100.0)**	**5,939,009**	**(100.0)**	**699,589**	**(100.0)**

[a] Sex was missing was 7205 children (England) with no missing data from Western Australia or Scotland.

[b] Deprivation scores were missing for 18,920 children (Western Australia), 146,588 (England) and 827 (Scotland).

Supplementary Table 2: Hospitalisation rate for ARI by diagnostic category by primary diagnosis only (PDx) and primary diagnosis plus all additional diagnoses (Any Dx) in infants aged <1 year and children aged 1-4 years in Western Australia, England and Scotland

	Western Australia			England			Scotland		
	Rate[a]		IRR (95% CI)	Rate[a]		IRR (95% CI)	Rate[a]		IRR (95% CI)
**ARI diagnosis**	**Any Dx**	**PDx**		**Any Dx**	**PDx**		**Any Dx**	**PDx**	
**<1 year[b]**									
Whooping cough	0.8	0.7	1.2 (1.0, 1.4)	0.5	0.5	1.1 (1.0, 1.2)	0.6	0.6	1.1 (0.9, 1.2)
Pneumonia	5.6	4.1	1.4 (1.3, 1.5)	3.8	2.9	1.3 (1.3, 1.3)	2.6	1.9	1.3 (1.2, 1.4)
Bronchiolitis	36.6	34.4	1.1 (1.0, 1.1)	34.2	32.2	1.1 (1.1, 1.1)	35.4	34.3	1.0 (1.0, 1.1)
Influenza	1.6	1.3	1.3 (1.1, 1.4)	0.4	0.3	1.4 (1.3, 1.5)	0.9	0.7	1.3 (1.2, 1.5)
Unspecified ARI	3.9	2.6	1.5 (1.4, 1.6)	5.9	4.6	1.3 (1.3, 1.3)	3.6	2.8	1.3 (1.2, 1.4)
Bronchitis	0.7	0.5	1.3 (1.1, 1.6)	0.5	0.4	1.2 (1.1, 1.3)	0.5	0.4	1.2 (1.1, 1.5)
Total ARI	46.4	43.7	1.1 (1.0, 1.1)	43.4	40.1	1.1 (1.1, 1.1)	42.1	40.7	1.0 (1.0, 1.1)
**1-4 years[c]**									
Whooping cough	0.05	0.04	1.1 (0.7, 1.9)	0.009	0.008	1.1 (0.9, 1.5)	0.02	0.01	1.1 (0.7, 2.0)
Pneumonia	4.3	3.7	1.2 (1.1, 1.2)	2.8	2.5	1.1 (1.1, 1.1)	2.3	2.1	1.1 (1.1, 1.1)
Bronchiolitis	2.7	2.3	1.2 (1.1, 1.2)	0.8	0.7	1.1 (1.1, 1.2)	2.0	1.9	1.1 (1.0, 1.1)
Influenza	0.6	0.4	1.3 (1.1, 1.4)	0.2	0.2	1.3 (1.2, 1.4)	0.3	0.2	1.2 (1.1, 1.4)
Unspecified ARI	3.0	2.2	1.4 (1.3, 1.5)	5.1	4.2	1.2 (1.2, 1.2)	3.9	3.3	1.2 (1.1, 1.2)
Bronchitis	0.3	0.2	1.4 (1.2, 1.7)	0.1	0.1	1.2 (1.1, 1.3)	0.1	0.1	1.3 (0.0, 1.6)
Total ARI	10.5	9.0	1.2 (1.1, 1.2)	8.7	7.6	1.1 (1.1, 1.1)	8.4	7.6	1.1 (0.0, 1.1)

IRR, incidence rate ratio

[a]Rate is per 1000/child-years

[b]2001-2012 for Western Australia and Scotland; 2004-2012 for England

[c]2005-2012 for Western Australia and Scotland; 2008-2012 for England
