# Supplementary material for: Temporal trends and socioeconomic differences in acute respiratory infection hospitalisations in children: an intercountry comparison of birth cohort studies in Western Australia, England and Scotland
Source: BMJ Open. 2019 May 19;9(5):e028710. doi: 10.1136/bmjopen-2018-028710 (PMC6530403; doi:10.1136/bmjopen-2018-028710)
Supplement: Author's manuscript [file bmjopen-2018-028710.draft_revisions.pdf]

BMJ Open is committed to open peer review. As part of this commitment we make the peer review history of every article we publish publicly available.

When an article is published we post the peer reviewers' comments and the authors' responses online. We also post the versions of the paper that were used during peer review. These are the versions that the peer review comments apply to.

The versions of the paper that follow are the versions that were submitted during the peer review process. They are not the versions of record or the final published versions. They should not be cited or distributed as the published version of this manuscript.

BMJ Open is an open access journal and the full, final, typeset and author-corrected version of record of the manuscript is available on our site with no access controls, subscription charges or pay-per-view fees (http://bmjopen.bmj.com).

If you have any questions on BMJ Open's open peer review process please email info.bmjopen@bmj.com

BMJ Open

Temporal trends and socio-economic differences in acute respiratory infection hospitalisations in children: an inter-country comparison of birth cohort studies in Western Australia, England and Scotland

Journal:	*BMJ Open*
Manuscript ID	bmjopen-2018-028710
Article Type:	Research
Date Submitted by the Author:	21-Dec-2018

Complete List of Authors:	Moore, Hannah; Wesfarmers Centre of Vaccines and Infectious Diseases, Telethon Kids Institute, The University of Western Australia, de Klerk, Nicholas; Telethon Kids Institute, Wesfarmers Centre of Vaccines and Infectious Diseases Blyth, Christopher C.; Division of Paediatrics, School of Medicine, The University of Western Australia; PathWest Laboratory Medicine WA, Perth Children's Hospital Gilbert, Ruth; University College London, Institute of Child Health Fathima, Parveen; Wesfarmers Centre of Vaccines and Infectious Diseases, Telethon Kids Institute, The University of Western Australia Zylbersztejn, Ania; University College London Great Ormond Street Institute of Child Health , Population, policy and practice Verfürden, Maximiliane ; University College London, Great Ormond Street Institute of Child Health Hardelid, Pia; UCL Institute of Child Health, Centre for Paediatric Epidemiology and Biostatistics

Keywords:	Respiratory infections < THORACIC MEDICINE, socio-economic disparity, infant, hospitalisation, record linkage

SCHOLARONE™
Manuscripts

Temporal trends and socio-economic differences in acute respiratory infection hospitalisations in children: an inter-country comparison of birth cohort studies in Western Australia, England and Scotland

Hannah C Moore[1], Nicholas de Klerk[1], Christopher C Blyth[1, 2, 3, 4], Ruth Gilbert[5], Parveen Fathima[1], Ania Zylbersztejn[5], Maximiliane Verfürden[5], Pia Hardelid[5]

[1]Wesfarmers Centre of Vaccines & Infectious Diseases, Telethon Kids Institute, The University of Western Australia, Perth, Australia

[2]School of Medicine, The University of Western Australia, Perth, Australia

[3]Department of Infectious Diseases, Princess Margaret Hospital for Children, Perth, Australia

[4]PathWest Laboratory Medicine WA, QE11 Medical Centre, Perth, Australia

[5]University College London Great Ormond Street Institute of Child Health, London, United Kingdom

Author for Correspondence:

Hannah C Moore, Wesfarmers Centre of Vaccines & Infectious Diseases, Telethon Kids Institute, The University of Western Australia. PO Box 855, West Perth, Western Australia 6872

Email: hannah.moore@telethonkids.org.au

Phone: + 61 8 6319 1427 / +61 409 100 007

For peer review only - http://bmjopen.bmj.com/site/about/guidelines.xhtml

ABSTRACT

Objectives: Acute respiratory infections (ARI) are a global cause of childhood morbidity. We compared temporal trends and socioeconomic disparities for ARI hospitalisations in young children across Western Australia, England and Scotland.

Design: Retrospective population-based cohort studies using linked birth, death and hospitalisation data.

Setting and Participants: Population birth cohorts spanning 2000-2012 (Western Australia and Scotland) and 2003-2012 (England).

Outcome Measures: ARI hospitalisations in infants (<12 months) and children (1-4 years) were identified through ICD-10 diagnosis codes. We calculated admission rates per 1000 child-years by diagnosis and jurisdiction- specific socio-economic deprivation and used negative binomial regression to assess temporal trends.

Results: The overall infant ARI admission rate was 44.3/1000 child-years in Western Australia, 40.7/1000 in Scotland and 40.1/1000 in England. Equivalent rates in children aged 1-4 years were 9.0, 7.6, and 7.6. Bronchiolitis was the most common diagnosis. Compared with the least socio-economically deprived, those most deprived had higher ARI hospitalisation risk (incidence rate ratio 3.9 [95% confidence interval 3.5, 4.2] for Western Australia; 1.9 [1.7, 2.1] for England; 1.3 [1.1, 1.4] for Scotland). ARI admissions in infants were stable in Western Australia but increased annually in England (5%) and Scotland (3%) after adjusting for non-ARI admissions, sex, and deprivation.

Conclusions: Admissions for ARI were higher in Western Australia and displayed greater socio-economic disparities than England and Scotland, where ARI rates are increasing. Prevention programs focusing on disadvantaged populations in all three countries are likely to translate into real improvements in the burden of ARI in children.

For peer review only - http://bmjopen.bmj.com/site/about/guidelines.xhtml

For peer review only

Keywords (3-10)

Acute respiratory infections; hospitalisation; socio-economic disparity; international comparison; infant; population; record linkage

ARTICLE SUMMARY

Strengths and limitations of this study

- We used population-level data from three countries to assess hospitalisation rates and changes over time in acute respiratory infections in children
- Analysis protocols and diagnosis coding was standardised across each country
- To control for changing admission thresholds within each country, we adjusted our models for all non-acute respiratory infection admissions
- The study provides insights into the preventable burden of acute respiratory infections
- A limitation of this study is the different measures of socio-economic deprivation available across the three countries

For peer review only

BACKGROUND

Acute respiratory infections (ARI) including bronchiolitis, pneumonia and influenza are a major cause of hospitalisation in children worldwide, responsible for approximately 12 million annual episodes in children under 5 years of age[1, 2]. In England, the hospital admission rate for ARI increased by 40% from 1999-2010 among children aged less than 15 years[3] and bronchiolitis was the most common reason for unplanned admissions in infants from 2010-2013.[4] While hospitalisations for ARI doubled from 1992-2000 in Western Australia,[5] they since stabilised 2000-2005.[6] Vaccination programmes including influenza, pertussis and pneumococcal disease have been implemented in North America, Europe and Australia, but the majority of ARI hospitalisations in high income countries are now caused by non-vaccine preventable viruses including Respiratory Syncytial Virus (RSV), Parainfluenza virus and Human Metapneumovirus.[7]

ARI hospitalisations are more common among children from poorer socio-economic backgrounds.[8, 9] In addition to access to inadequate health care, risk factors for developing severe symptoms of ARIs, including prematurity, low birth weight, congenital anomalies, exposure to environmental tobacco smoke, damp and mould, and household overcrowding are all more common among children growing up in more deprived families in both high and low income settings.[10, 11] Understanding the impact of socio-economic disparities on ARI hospitalisations among children (both over time and between countries) can provide an estimate of the preventable proportion of ARI. Linkage of administrative health datasets provides a platform to investigate these trends in populations over many years. Additionally, the availability of comparable hospital admission datasets with similar coding systems using International Classification of Diseases, 10th edition (ICD-10) diagnosis codes allows comparison of hospitalisation rates among children for ARI according to deprivation level.

For peer review only - http://bmjopen.bmj.com/site/about/guidelines.xhtml

Using record linkage resources within Western Australia, England and Scotland, we conducted a comparative analysis of the three jurisdictions to investigate the hospitalisation rates for ARI in children aged less than 5 years. All three jurisdictions have publicly funded health care with free access to primary and public hospital care. Our aim was to compare population-based hospitalisation rates by ARI diagnosis, age and level of socio-economic deprivation, and assess how ARI hospitalisation rates have changed over time.

METHODS

Data Sources and Study Populations

We conducted separate population-based birth cohort studies using administrative data from Western Australia, England and Scotland. Western Australia covers the western third of Australia, an area of 2.5 million square kilometres with a population of nearly 2.6 million,[12] 3.6% of whom identify as being Aboriginal and/or Torres Strait Islander (herein referred to as Aboriginal).[13] Births were identified from the Midwives' Notification System and Birth Register, deaths were identified from the Death Register and hospitalisations were recorded in the Hospital Morbidity Database Collection that provides full coverage of all hospital separations (hereafter referred to as hospitalisations). Data were extracted and probabilistically linked by the Western Australian Data Linkage Branch using a series of demographic identifiers.[14] England has a population of 53.9 million.[15] The birth cohort was established by linking hospital birth and delivery records from the Hospital Episode Statistics (HES) database.[16] Hospitalisations and deaths were identified via linkage to mortality registration data from the Office for National Statistics.[17] Data linkage in England was carried out by NHS Digital, using a deterministic algorithm based on the NHS number (a unique patient identifier in the English NHS), postcode, date of birth, sex and local hospital numbers.

For peer review only - http://bmjopen.bmj.com/site/about/guidelines.xhtml

Scotland has a population of 5.3 million.[15] The Scottish birth cohort was developed through linking data from birth registration and maternity databases[18, 19]. Hospitalisations and deaths were identified via linkage to the Scottish Morbidity Record 01 (SMR-01) and mortality records using deterministic linkage carried out by the electronic Data Research and Innovation Service (eDRIS) based on the Community Health Index number, a unique identifier recorded on all births and subsequent encounters within the Scottish NHS.

The datasets represented 99.9% of all births in Western Australia, 97.5% in England[20] and 100% in Scotland with full coverage of inpatient and day admissions. Our study population comprised of singleton births in Western Australia and Scotland 2000-2012 and England 2003-2012. Multiple births were excluded due to a higher likelihood of linkage error. Children were followed from birth until their fifth birthday, date of death, or 30 June 2013 (the end of follow-up) or (Scotland only) date of emigration, whichever occurred first.

Outcome Measures

Our outcome measure was an ARI emergency hospitalisation for children in their first 5 years of life. All inter-hospital transfers were collapsed into a single admission. We identified hospitalisations for ARI using a selection of ICD-10 diagnosis codes (ICD-10-AM for Western Australia).[21] Hospitalisation data for each jurisdiction provided a principal diagnosis code and up to 20 secondary diagnosis codes in Western Australia, 19 in England and 5 in Scotland. We identified ARI hospitalisations using the principal diagnosis code and all the available additional diagnosis codes as six diagnostic groups: whooping cough (A37), influenza (J09-J11), pneumonia (J12-J18, B59, B05.2, B37.1, B01.2), bronchitis (J20, J40), bronchiolitis (J21) and unspecified acute lower respiratory infection.(J22) Consistent with our previous Western Australian work,[6] ARI hospitalisations within

For peer review only - http://bmjopen.bmj.com/site/about/guidelines.xhtml

For peer review only

14 days of a previous ARI hospitalisation were classified as a single infection episode. In such cases we applied a hierarchical diagnosis algorithm[6] within the readmission set in order to code an overall principal diagnosis. This algorithm ranked diagnoses in order of disease severity: whooping cough, pneumonia, bronchiolitis, influenza, unspecified ALRI and bronchitis. Children with missing data on sex or deprivation were excluded from the analyses.

Exposure Measures

We assessed hospitalisations for ARI in infants aged less than 12 months and young children aged 1-4 years at time of admission. Other exposure measures of interest were sex, level of socio-economic deprivation and admission year. In Western Australia, socio-economic deprivation was measured through the Index of Relative Disadvantage (IRSAD), one of the four Socio-Economic Indexes for Areas (SEIFA) derived by the Australian Bureau of Statistics.[22] The IRSAD score is derived from 17 different variables including low income, internet connection, unemployment and education.[22] Scores were grouped into Collectors District, the smallest unit for population-based analyses which, on average, consist of approximately 200 dwellings. For England, socio-economic deprivation was measured through the Index of Multiple Deprivation (IMD), based on seven domains of deprivation including income, employment, education, crime, barriers to housing and living environment.[23] IMD scores are measured at Lower Super Output Area Level, covering an average of 1200-1500 households. For Scotland, deprivations scores were based on the Carstairs Index, based on four variables including car ownership, male unemployment, overcrowding and low occupational social class. The Carstairs Index is measured at postcode sector level, which contains an average of 5000 people[24] In all jurisdictions socio-economic deprivation scores were based on mother's residential address at time of her child's delivery and were grouped into four levels based on a country level ranking with the lowest scores representing the most socio-economically deprived.

For peer review only - http://bmjopen.bmj.com/site/about/guidelines.xhtml

Statistical Analysis

Consistent methodology was applied to the assembled datasets in the three jurisdictions. We calculated hospitalisation rates per 1000 child-years at risk for each diagnostic grouping of ARI (as principal diagnosis). To assess the impact of including additional diagnosis codes, we compared hospitalisation rates derived using the principal diagnosis code only with rates derived from using the principal plus all additional diagnosis codes (any diagnosis). We used any diagnosis to assess ARI rates by socio-economic deprivation and year of admission. We present age-specific hospitalisation rates with 95% confidence intervals (CI) and where appropriate, rates were compared using incidence rate ratios (IRRs) with 95% CIs. To assess temporal trends, we plotted annual hospitalisation rates in the two age groups for each jurisdiction by admission year for all ARIs and bronchiolitis, pneumonia and unspecified ALRI's. We also used negative binomial regression models to assess linear temporal trends in infant hospitalisations from 2001-2012 (Western Australia and Scotland) and 2004-2012 (England). Year of admission was included as a linear term in the models, and the natural logarithm of child-years at risk was included as an offset in the models. Trends over time in ARI admission rates were assumed to be statistically significant if the Wald test *p*-value for the coefficient for the linear year term was <0.05. Models were adjusted for sex and the 4-level socioeconomic indicator and we present IRR's with 95% CI's. In order to control for overall trends in hospitalisation we also adjusted the models for the number of all non-ARI emergency admissions.[25] All data analyses were conducted within each jurisdiction in Stata version 14.0.[26]

Public and Patient Involvement

A community reference group located in Western Australia was consulted during the conduct of this study. No individual patients were involved.

For peer review only - http://bmjopen.bmj.com/site/about/guidelines.xhtml

RESULTS

A total of 337,909 (Western Australia), 5,939,009 (England) and 699,590 (Scotland) births were included in the study (Supplementary Table 1). There were 14,480 infant hospitalisations for ARI as a principal diagnosis in Western Australia, 217,985 for England and 26,103 for Scotland giving overall infant hospitalisation rates of 44.3/1000 child-years for Western Australia, 40.7/1000 for Scotland and 40.1/1000 for England. In all jurisdictions, bronchiolitis had the highest hospitalisation rates accounting for 79% of ARI admissions in infants in Western Australia, 79% in England, and 84% in Scotland (Table 1). ARI hospitalisation rates in infants were higher in Western Australia compared with England and Scotland across all ARI diagnoses, most notably for pneumonia, where rates were 1.4-2.2 times higher compared to England and Scotland. The only exception was for unspecified ALRI where the hospitalisation rate in infants were 70% higher in England than in Scotland and Western Australia. ARI hospitalisation rates in children aged 1-4 years were 19% higher in Western Australia compared with England and Scotland (Table 1). The most common ARI principal diagnosis among children aged 1-4 years was pneumonia in Western Australia (42%) and unspecified ALRI in England (54.6%) and Scotland (43.9%). Consequently, hospitalisation rates for pneumonia in Western Australian children aged 1-4 years were 1.5-1.8 times higher than England and Scotland.

When ARI hospitalisations were identified based on any diagnosis compared with principal diagnosis only, the difference in hospitalisation rates varied across diagnoses with the most notable difference for unspecified ALRI in Western Australia where rates were 1.5 (95% CI: 1.4, 1.6) times higher in infants when using any diagnosis compared with principal diagnosis only (Supplementary Table 2).

ARI hospitalisation rates were higher for children from the most socio-economically deprived areas. The association with deprivation was greatest in Western Australia and more marked in infants

For peer review only - http://bmjopen.bmj.com/site/about/guidelines.xhtml

compared to young children aged 1-4 years (Figure 1). The relative difference in ARI hospitalisation rates between the most and least deprived infants was 3.5 (95% CI: 3.2, 3.7) in Western Australia; 1.8 for England and 1.3 for Scotland with similar patterns in children aged 1-4 years (Figure 1). In multivariable models, level of socio-economic deprivation was significantly associated with all ARI categories in all infants but most notably in Western Australia, and in particular, pneumonia (IRR 6.9, 95% CI: 5.6, 8.6) and unspecified ALRI (IRR 8.9, 95% CI: 6.7, 11.8; Table 2).

Overall, ARI hospitalisation rates have increased in England and Scotland, but declined (infants) or remained stable (children aged 1-4 years) in Western Australia (Figure 2). After adjusting for sex, deprivation and non-ARI emergency hospitalisations, the ARI hospitalisation rate among infants increased by 5% per year in England (IRR 1.05, 95% CI: 1.04, 1.07) and by 3% per year (IRR 1.03, 95% CI: 1.02, 1.04) in Scotland with no statistically significant trend in Western Australia (IRR 0.99, 95% CI: 0.98, 1.00; Table 2, Figure 2). Similar results were seen for bronchiolitis admissions in infants.

Diverging trends were seen with pneumonia and unspecified ALRI across the three jurisdictions with pneumonia hospitalisation rates in infants declining in Western Australia from 9.0/1000 in 2002 to 3.9/1000 in 2012 while rates remained steady around 3-4/1000 in England and 2-3/1000 in Scotland (Figure 2). After adjusting for sex, socio-economic deprivation and non-ARI admissions, the annual decline in pneumonia hospitalisations was 6% in Western Australia (IRR 0.94, 95%CI: 0.93, 0.96), 2% in England and 3% in Scotland (Table 2). Unspecified ALRI declined in Western Australia annually by 5% but increased by 6% and 2% annually in England and Scotland (Table 2).

For peer review only - http://bmjopen.bmj.com/site/about/guidelines.xhtml

DISCUSSION

ARI, particularly bronchiolitis, continues to be an important cause of infant and childhood hospitalisation. The availability of linked administrative data in three economically similar jurisdictions with publicly funded healthcare systems afforded us the opportunity to compare ARI hospitalisation rates in children. Overall, admission rates were highest in Western Australia and decreasing or remaining stable but increasing in England and Scotland. The relative differences in ARI admission rates between children from the most socioeconomically deprived areas to the least deprived areas were largest in Western Australia.

The interpretation of hospitalisation trends across countries is complex. We have found higher rates of ARI admissions in Western Australia compared with England and Scotland which could mean a higher incidence in ARI, a higher risk of developing more severe symptoms, or differences in diagnostic coding or hospital admission thresholds. A recent study comparing admission rates between England and Ontario finding substantially higher rates in England was partly explained by differing admission thresholds from differential waiting practices and policies in emergency departments.[4] Comparisons of asthma admissions from national hospital data in Finland and Sweden noted diverging trends citing differences in national coding guidelines and subsequent altered admission thresholds.[27] In an attempt to control for changing admissions thresholds over time within each jurisdiction, we adjusted our multivariable models for the overall trend in non-ARI emergency hospital use. However we could not adjust for differing thresholds between countries. Emergency hospitalisations are increasing at a faster rate in England compared to other parts of the United Kingdom[28] and our data here suggests that hospitalisations due to unspecified ALRI and bronchiolitis in England are contributing to that increase. It is also possible that diverging trends are a result of diagnostic shifts in that for the same clinical presentations, a diagnosis of unspecified ALRI is given in England while other non-specific codes (including codes we have not assessed) are given

For peer review only - http://bmjopen.bmj.com/site/about/guidelines.xhtml

in Western Australia and Scotland. The use of additional diagnosis codes for ARI seemed more frequent in Western Australia compared with England and Scotland and should be taken into consideration for future comparative studies using ICD diagnosis codes.

Hospitalisation rates for ARI were significantly associated with level of socio-economic deprivation, consistent with an earlier analysis in England.[29] This association was strongest in Western Australia with IRRs for those in the most deprived level in the order of 3.9 for all ARIs, up to 8.9 for unspecified ALRI. There appeared a linear relationship with level of deprivation and rates of ARI in Western Australia while rates in all levels (bar the most deprived) not differing in England and Scotland. Western Australian data were inclusive of Aboriginal children, an Indigenous population with higher levels of socio-economic disadvantage[30] compared to their non-Aboriginal peers and a significantly higher burden of pneumonia worldwide,[6, 31, 32] despite reductions in the 2000's and further reductions seen in our results here, most likely due to the positive impact of pneumococcal vaccination.[6, 33] This most likely explains the higher rates of pneumonia seen in Western Australia compared with England and Scotland. Aboriginal children also suffer a disproportionate burden of RSV,[34] the major cause of bronchiolitis which could explain the higher bronchiolitis rates in Western Australia than in England and Scotland. However level of socio-economic deprivation has been associated with hospitalisations for respiratory infections in both Aboriginal and non-Aboriginal children[9] so the contribution of Aboriginal children alone cannot explain the higher socio-economic disparities seen here. Indeed, when Aboriginal children were removed from the analysis, the socio-economic disparities remained, although slightly lessened, and were still higher than England and Scotland (e.g. the IRR for most deprived children for all ARI reduced from 3.9 to 2.1 and for unspecified ALRI reduced from 8.9 to 2.9 (data not shown)). Respiratory infections continue to be a source of health inequalities among disadvantaged children worldwide. Geographical

For peer review only - http://bmjopen.bmj.com/site/about/guidelines.xhtml

remoteness is more of an issue in Western Australia due to its sheer geographical size in comparison to England and Scotland. The lack of adequate primary care in rural and remote Australia[35] which is often coupled with lower socio-economic levels could be driving higher hospitalisation rates. Nevertheless, these important findings highlight the need for targeted prevention programs such as smoking cessation, improved housing and timely vaccination for key respiratory pathogens for the most disadvantaged populations in all three jurisdictions.

Unlike the United Kingdom, Australia does not have a uniform policy for seasonal influenza vaccination. Relative to other ARI diagnoses, recorded influenza hospitalisation rates are low. Assessing the impact of the universal childhood vaccination program for influenza in the United Kingdom introduced in 2013-14 is likely to be challenging without linking national-level birth cohorts to infection surveillance data. This has already been implemented in Scotland.[36] There is also renewed interest in preventing morbidity due to RSV with vaccination.[37] Understanding the baseline hospitalisation rates for RSV-bronchiolitis and pneumonia prior to when vaccination is available is critical to aid in implementation and for its ongoing evaluation post implementation.

We conducted our analysis on near total population birth cohorts in each jurisdiction and thus our outcome measures have narrow confidence intervals and minimum selection bias. An additional strength of the population-based cohort design is standardisation of analysis protocols and the provision of large numbers allowing us to assess temporal trends and associations with less common infections. The hospital morbidity database systems used in all three jurisdictions have the same population coverage of all inpatient admissions and day surgeries further adding to the validity of our estimates.

For peer review only - http://bmjopen.bmj.com/site/about/guidelines.xhtml

However, our study does have some limitations. The socio-economic deprivation scores used were jurisdiction specific and included different items to represent disadvantage. In addition, area-level socio-economic deprivation was only measured at birth. Therefore, the observed association between area-level socio-economic deprivation and the rate of ARI admissions may be subject to increasing measurement error as the child's age increases. How socio-economic deprivation is associated with morbidity due to ARI at the primary care level is unknown but perhaps likely to aid in explaining disparities in socio-economic deprivation that we have seen here. While primary care data is more readily available in England and Scotland, limited data with adequate diagnostic information is available for population-based studies in Western Australia. As previously alluded to, there also may be differences in admission thresholds across the three jurisdictions that may explain some higher admission rates across countries. A comparison of emergency department presentations in conjunction with hospitalisations for ARI could be useful here, although diagnostic information from emergency department data is limited[38] and no individual level data on emergency department visits exist in Scotland. Additionally, through our experience of linking routine laboratory data to hospital data in Western Australia, we are aware of unspecific ICD codes that are associated with detections of respiratory viral pathogens.[39] We did not include such ICD codes (e.g. viral infection of unspecified site "B34") in this analysis. However we would not expect the potential exclusion of ARI hospitalisations to alter the direction of our results in terms of association with socio-economic deprivation.

CONCLUSIONS

Population-based administrative data from economically similar developed countries provides a powerful tool to conduct international comparative studies that can compare and contrast the epidemiology of, and healthcare responses to, respiratory infections. Western Australia experiences higher admissions in children for ARI and a greater disparity in rates according to level of socio-

For peer review only - http://bmjopen.bmj.com/site/about/guidelines.xhtml

economic deprivation. Rates are overall slightly lower in England and Scotland but are increasing, particularly in England. These findings suggest that prevention programs focusing on disadvantaged populations in all three countries are likely to translate into real improvements in the burden of ARI in children. We are planning to use these administrative data to assess effectiveness of interventions (such as vaccination) and how this may affect disparities in ARI admissions rates according to socio-economic deprivation.

Acknowledgements

We would like to acknowledge the Western Australian Data Custodians, and particularly Alexandra Merchant and Mikhalina Dombrovskaya from the Western Australian Data Linkage Branch, for their assistance and support in collating the data for Western Australia.

List of Abbreviations

ALRI: Acute lower respiratory infection; ARI: Acute respiratory infection; CI: Confidence interval; HES: Hospital Episode Statistics; ICD: International Classification of Diseases; IMD: Index of Multiple Deprivation; IRR: Incidence rate ratio; IRSAD: Index of Relative Disadvantage; NHS: National Health Service; RSV: Respiratory syncytial virus; SEIFA: Socio-Economic Index for Australia

Competing interests

The authors do not have any commercial or other association that might pose a conflict of interest.

For peer review only - http://bmjopen.bmj.com/site/about/guidelines.xhtml

Funding

This work was supported by a National Health and Medical Research Council Project Grant [1045668], a University of Western Australia Research Collaboration Award [to HCM] and a Wesfarmers Centre of Vaccines & Infectious Diseases Seed Grant [to HCM, CCB, PH]. CCB and HCM are supported by National Health and Medical Research Council Fellowships [1034254 to HCM and 1111596 to CCB]. PH was funded by a National Institute for Health Research postdoctoral Fellowship, reference number PDF-2013–06–004. The views expressed in this publication are those of the author(s) and not necessarily those of the NHS, the National Institute for Health Research or the Department of Health. AZ's PhD studentship is funded by awards to establish the Farr Institute of Health Informatics Research, London, from the Medical Research Council, Arthritis Research UK, British Heart Foundation, Cancer Research UK, Chief Scientist Office, Economic and Social Research Council, Engineering and Physical Sciences Research Council, National Institute for Health Research, National Institute for Social Care and Health Research, and Wellcome Trust (grant MR/K006584/1). AZ is also supported by the Administrative Data Research Centre for England (funded by the Economic and Social Research Council). MV's PhD studentship is funded by the UBEL DTP of the Economic and Social Research Council. MV was also supported by the Policy Research Unit in the Health of Children, Young People and Families (reference 109/00017), which is funded by the Department of Health Policy Research Programme at UCL. This is an independent report commissioned and funded by the Department of Health. The views expressed are not necessarily those of the Department.

Ethics approval and consent to participate

Approval to use the Western Australian data was granted by the Western Australian Department of Health Human Research Ethics Committee, the Western Australian Aboriginal Health Ethics

For peer review only - http://bmjopen.bmj.com/site/about/guidelines.xhtml

Committee and the Western Australian Data Linkage Branch. Hospital Episode Statistics data for England can be accessed by researchers applying to the Health and Social Care Information Centre for England. For Scotland, approvals were obtained from the Public Benefit and Privacy Panel for Health and Social Care, reference number 1516-0405.

Consent for publication

Not applicable.

Data Sharing Statement

We cannot share the individual-level data used for this study under our agreements with the data providers. The datasets analysed during the current study can be applied for from the Western Australian Data Linkage System (Western Australia; http://www.datalinkage-wa.org.au/), NHS Digital; (England; http://content.digital.nhs.uk) and the electronic Data Research and Innovation Service (Scotland; http://www.isdscotland.org/Products-and-Services/eDRIS/). Derived data from these datasets for each jurisdiction are within the paper. No additional data are available.

Authors' contributions

HCM, CCB and PH conceived the study design. PF assisted with data cleaning and coding in Western Australia, AZ and MV assisted with data extraction for England and Scotland. Statistical analysis was conducted by HCM (Western Australia) and PH (England and Scotland) with expert advice from NdK with critical revisions for intellectual content from CCB and RG. HCM drafted the first manuscript with PH. All authors read and approved the final manuscript.

For peer review only - http://bmjopen.bmj.com/site/about/guidelines.xhtml

REFERENCES

1. Nair H, Simoes EA, Rudan I*, et al.* Global and regional burden of hospital admissions for severe acute lower respiratory infections in young children in 2010: a systematic analysis. *Lancet*. 2013;**381**:1380-90.

2. Shi T, McAllister DA, O'Brien KL*, et al.* Global, regional, and national disease burden estimates of acute lower respiratory infections due to respiratory syncytial virus in young children in 2015: a systematic review and modelling study. *Lancet*. 2017;**390**:946-58.

3. Gill PJ, Goldacre MJ, Mant D*, et al.* Increase in emergency admissions to hospital for children aged under 15 in England, 1999–2010: national database analysis. *Arch Dis Child*. 2013.

4. Harron K, Gilbert R, Cromwell D*, et al.* International comparison of emergency hospital use for infants: data linkage cohort study in Canada and England. *BMJ Quality & Safety*. 2017.

5. Moore H, Burgner D, Carville K*, et al.* Diverging trends for lower respiratory infections in non-Aboriginal and Aboriginal children. *J Paediatr Child Health*. 2007;**43**:451-7.

6. Moore HC, Lehmann D, de Klerk N*, et al.* Reduction in disparity for pneumonia hospitalisations between Australian Indigenous and non-Indigenous children. *Journal of epidemiology and community health*. 2012;**66**:489-94.

7. Anderson AJ, Snelling TL, Moore HC*, et al.* Advances in Vaccines to Prevent Viral Respiratory Illnesses in Children. *Paediatr Drugs*. 2017;**19**:523-31.

8. Cakmak S, Hebbern C, Cakmak JD*, et al.* The modifying effect of socioeconomic status on the relationship between traffic, air pollution and respiratory health in elementary schoolchildren. *J Environ Manage*. 2016;**177**:1-8.

9. Moore HC, de Klerk N, Richmond P*, et al.* A retrospective population-based cohort study identifying target areas for prevention of acute lower respiratory infections in children. *BMC Public Health*. 2010;**10**:757.

For peer review only - http://bmjopen.bmj.com/site/about/guidelines.xhtml

10. MacIntyre EA, Gehring U, Mölter A, *et al.* Air pollution and respiratory infections during early childhood: an analysis of 10 European birth cohorts within the ESCAPE Project. *Environ Health Perspect*. 2014;**122**:107.

11. Smith KR, Samet JM, Romieu I, *et al.* Indoor air pollution in developing countries and acute lower respiratory infections in children. *Thorax*. 2000;**55**:518-32.

12. ABS. 3101.0 - Australian Demographic Statistics, Jun 2014. Australian Bureau of Statistics; 2015; Available from: http://www.abs.gov.au/AUSSTATS/abs@.nsf/Lookup/3101.0Main+Features1Jun%202014?OpenDocument.

13. HealthInfoNet. What details do we know about the Indigenous population? : Australian Indigenous HealthInfoNet 2015; Available from: http://www.healthinfonet.ecu.edu.au/health-facts/health-faqs/aboriginal-population.

14. Holman CD, Bass AJ, Rosman DL, *et al.* A decade of data linkage in Western Australia: strategic design, applications and benefits of the WA data linkage system. *Aust Health Rev*. 2008;**32**:766-77.

15. Office for National Statistics. 2011 Census: Population Estimates for the United Kingdom, March 2011. 2012 [6 February 2018]; Available from: https://www.ons.gov.uk/peoplepopulationandcommunity/populationandmigration/populationestimates/bulletins/2011censuspopulationestimatesfortheunitedkingdom/2012-12-17.

16. Herbert A, Wijlaars L, Zylbersztejn A, *et al.* Data Resource Profile: Hospital Episode Statistics Admitted Patient Care (HES APC). *Int J Epidemiol*. 2017;**46**:1093-i.

17. NHS Digital. A Guide to Linked Mortality Data from Hospital Episode Statistics and the Office for National Statistics. 2015 [6 February 2018]; Available from: http://content.digital.nhs.uk/media/11668/HES-ONS-Mortality-Data-Guide/pdf/mortality_guide.pdf.

For peer review only - http://bmjopen.bmj.com/site/about/guidelines.xhtml

18. Administrative Data Liaison Service. Birth Registrations - National Records of Scotland. 2016 [4 November 2016]; Available from: http://www.adls.ac.uk/national-records-of-scotland/birth-registrations/?detail.

19. Administrative Data Liaison Service. SMR02 – Maternity Inpatient and Day Case dataset. 2016 [4 November 2016]; Available from: http://www.adls.ac.uk/nhs-scotland/maternity-inpatient-and-day-case-smr02/?detail.

20. Harron K, Gilbert R, Cromwell D*, et al.* Linking Data for Mothers and Babies in De-Identified Electronic Health Data. *PLoS ONE*. 2016;**11**:e0164667.

21. Roberts RF, Innes KC, Walker SM. Introducing ICD-10-AM in Australian hospitals. *The Medical Journal of Australia*. 1998;**169**:S32-5.

22. Pink B. An Introduction to Socio-Economic Indexes for Areas (SEIFA) - Information paper: Australian Bureau of Statistics, Commonwealth of Australia 2008.

23. Smith T, Noble M, Noble S*, et al.* The English Indices of Deprivation 2015: Technical Report: Department for Communities and Local Government 2015.

24. Brown D, Allik M, Dundas R*, et al.* Carstairs Scores for Scottish Postcode Sectors, Datazones & Output Areas from the 2011 Census: University of Glasgow 2014.

25. Gonzalez-Izquierdo A, Cortina-Borja M, Woodman J*, et al.* Maltreatment or violence-related injury in children and adolescents admitted to the NHS: comparison of trends in England and Scotland between 2005 and 2011. *BMJ Open*. 2014;**4**:e004474.

26. StataCorp. Stata Statistical Software. Release 14. College Station, TX.: StataCorp LP.; 2015.

27. Kivistö JE, Protudjer JLP, Karjalainen J*, et al.* Trends in paediatric asthma hospitalisations – differences between neighbouring countries. *Thorax*. 2017.

28. Department of Health. Emergecny admissions to hospital: managing the demand: National Audit Office 2013.

29. Hawker JI, Olowokure B, Sufi F*, et al.* Social deprivation and hospital admission for respiratory infection:: an ecological study. *Respir Med*. 2003;**97**:1219-24.

For peer review only - http://bmjopen.bmj.com/site/about/guidelines.xhtml

30. Zubrick SR, Lawrence D, Silburn S*, et al.* The Western Australian Aboriginal Child Health Survey: The Health of Aboriginal Children and Young People 1ed. Perth, Western Australia: Telethon Institute for Child Health Research 2004.

31. Holman RC, Hennessy TW, Haberling DL*, et al.* Increasing trend in the rate of infectious disease hospitalisations among Alaska Native people. *Int J Circumpolar Health*. 2013;**72**:20994.

32. O'Grady K-Ann F, Lee Katherine J, Carlin John B*, et al.* Increased risk of hospitalization for acute lower respiratory tract infection among Australian Indigenous infants 5-23 months of age following pneumococcal vaccination: A cohort study. *Clin Infect Dis*. 2010;**50**:970-8.

33. Jardine A, Menzies RI, McIntyre PB. Reduction in Hospitalizations for Pneumonia Associated With the Introduction of a Pneumococcal Conjugate Vaccination Schedule Without a Booster Dose in Australia. *Pediatr Infect Dis J*. 2010;**29**:000-.

34. Moore HC, Lim FJ, Fathima P*, et al.* Exploring RSV epidemiology in Australian chidlren: estimates of the population burden and effectiveness of a maternal vaccine. **RSV Vaccines for the World**; 29 Nov - 1 Dec; Malaga, Spain2017.

35. Thomas SL, Wakerman J, Humphreys JS. Ensuring equity of access to primary health care in rural and remote Australia - what core services should be locally available? *Int J Equity Health*. 2015;**14**:111.

36. Hardelid P, Verfuerden M, McMenamin J*, et al.* Risk factors for admission to hospital with laboratory-confirmed influenza in young children: birth cohort study. *Eur Respir J*. 2017;**50**:1700489.

37. World Health Organization. RSV Vaccine Research and Develoment Technology Roadmap2017.

38. Moore HC, de Klerk N, Jacoby P*, et al.* Can linked emergency department data help assess the out-of-hospital burden of acute lower respiratory infections? A population-based cohort study. *BMC Public Health*. 2012;**12**:703.

39. Lim F, Blyth CC, Fathima P*, et al.* Record linkage study of the pathogen-specific burden of respiratory viruses in children. *Influenza & Other Respiratory Viruses*. 2017;**11**:502-10.

For peer review only - http://bmjopen.bmj.com/site/about/guidelines.xhtml

Figure legends

Figure 1: Hospitalisation rates for ARI in Western Australia, England and Scotland by level of socio-economic deprivation for A) infants (<1 year) and B) young children (1-4 years). Those in the <10% level represent the most deprived and those ≥ 90% represent those least deprived.

Figure 2: Hospitalisation rates by year of admission for infants (<1 year) and children (1-4 years) in Western Australia, England and Scotland for ARI, bronchiolitis, pneumonia and unspecified ALRI.

For peer review only

Table 1: Number of admissions and hospitalisation rate for ARI by diagnostic category by principal diagnosis in infants aged <1 year and children aged 1-4 years in Western Australia, England and Scotland

Diagnosis	Western Australia				England				Scotland			
	n	(%)	Rate[a]	(95% CI)	n	(%)	Rate[a]	(95% CI)	n	(%)	Rate[a]	(95% CI)
**<1 year[b]**												
Whooping cough	220	(1.6)	0.7	(0.6, 0.8)	2395	(1.1)	0.5	(0.4, 0.5)	372	(1.4)	0.6	(0.5, 0.6)
Pneumonia	1278	(9.4)	4.1	(3.9, 4.4)	15592	(7.2)	2.9	(2.9, 3.0)	1245	(4.8)	1.9	(1.8, 2.1)
Bronchiolitis	10,652	(78.7)	34.4	(33.8, 35.1)	171805	(78.8)	32.2	(32.1, 32.4)	22021	(84.4)	34.3	(33.9, 34.8)
Influenza	407	(3.0)	1.3	(1.2, 1.4)	1627	(0.7)	0.3	(0.3, 0.3)	426	(1.6)	0.7	(0.6, 0.7)
Unspecified ALRI	809	(6.0)	2.6	(2.4, 2.8)	24563	(11.3)	4.6	(4.5, 4.7)	1797	(6.9)	2.8	(2.7, 2.9)
Bronchitis	169	(1.2)	0.5	(0.5, 0.6)	2003	(0.9)	0.4	(0.4, 0.4)	242	(0.9)	0.4	(0.3, 0.4)
All ARI	13,535	(100.0)	43.7	(43.0, 44.5)	217985	(100.0)	40.1	(40.7, 41.1)	26103	(100.0)	40.7	(40.2, 41.2)
**1-4 years[c]**												
Whooping cough	33	(0.4)	0.04	(0.03, 0.06)	95	(0.1)	0.008	(0.007, 0.01)	23	(0.2)	0.01	(0.01,0.02)
Pneumonia	3031	(41.6)	3.7	(3.6, 3.9)	29741	(33.2)	2.5	(2.5, 2.6)	3411	(26.9)	2.1	(2.0, 2.1)
Bronchiolitis	1893	(26.0)	2.3	(2.2, 2.4)	8283	(9.2)	0.7	(0.7, 0.7)	3141	(24.7)	1.9	(1.8, 2.0)
Influenza	366	(5.0)	0.4	(0.4, 0.5)	1714	(1.9)	0.2	(0.1, 0.2)	392	(3.1)	0.2	(0.2, 0.3)
Unspecified ALRI	1767	(24.3)	2.2	(2.1, 2.3)	48910	(54.6)	4.2	(4.1, 4.2)	5570	(43.9)	3.3	(3.3, 3.4)
Bronchitis	195	(2.7)	0.2	(0.2, 0.3)	859	(1.0)	0.1	(0.1, 0.1)	161	(1.3)	0.1	(0.1, 0.1)
All ALRI	7285	(100.0)	9.0	(8.8, 9.2)	89602	(100.0)	7.6	(7.6, 7.7)	12698	(100.0)	7.6	(7.5, 7.8)

[a]Rate is per 1000/child-years

[b]2001-2012 for Western Australia and Scotland; 2004-2012 for England

For peer review only

[c]2005-2012 for Western Australia and Scotland; 2008-2012 for England

For peer review only

For peer review only - http://bmjopen.bmj.com/site/about/guidelines.xhtml

Table 2: Risk of hospitalisation for bronchiolitis, pneumonia, unspecified ALRI and overall ARI from log-linear modelling in infants aged <1 year in Western Australia, England and Scotland

Exposure	Western Australia		England		Scotland	
	IRR	(95% CI)	IRR	(95% CI)	IRR	(95% CI)
**Bronchiolitis**						
Year[a]	0.99	(0.98, 1.00)	1.05	(1.04, 1.07)	1.04	(1.03, 1.05)
Male	*Reference*		*Reference*		*Reference*	
Female	0.68	(0.64, 0.72)	0.68	(0.63, 0.74)	0.70	(0.64, 0.77)
Deprivation <10%	3.34	(3.02, 3.71)	1.94	(1.73, 2.19)	1.28	(1.16, 1.42)
Deprivation 10-49%	2.04	(1.75, 2.37)	1.48	(1.07, 2.06)	1.29	(0.89, 1.87)
Deprivation 50-89%	1.36	(1.19, 1.55)	1.18	(0.95, 1.45)	1.09	(0.85, 1.40)
Deprivation ≥90%	*Reference*		*Reference*		*Reference*	
Non-ARI admissions	1.00	(1.00, 1.00)	1.00	1.00, 1.00)	1.00	(1.00, 1.00)
**Pneumonia**						
Year[a]	0.94	(0.93, 0.96)	0.98	(0.97, 0.99)	0.97	(0.94, 0.99)
Male	*Reference*		*Reference*		*Reference*	
Female	0.75	(0.67, 0.84)	0.76	(0.70, 0.82)	0.80	(0.67, 0.97)
Deprivation 0-10%	6.91	(5.59, 8.56)	1.47	(1.30, 1.66)	1.09	(0.88, 1.37)
Deprivation 10-49%	3.26	(2.49, 4.28)	0.90	(0.65, 1.25)	0.74	(0.39, 1.43)
Deprivation 50-89%	1.66	(1.29, 2.13)	0.86	(0.70, 1.07)	0.80	(0.51, 1.25)
Deprivation >90%	*Reference*		*Reference*		*Reference*	
Non-ARI admissions	1.00	(1.00, 1.00)	1.00	(1.00, 1.00)	1.00	(1.00, 1.00)
**Unspecified ALRI**						
Year[a]	0.95	(0.93, 0.97)	1.06	(1.05, 1.07)	1.02	(1.00, 1.04)
Male	*Reference*		*Reference*		*Reference*	
Female	0.62	(0.54, 0.71)	0.65	(0.61, 068)	0.73	(0.62, 0.85)
Deprivation <10%	8.90	(6.69, 11.83)	1.81	(1.66, 1.98)	0.93	(0.78, 1.12)
Deprivation 10-49%	4.18	(2.93, 5.96)	1.34	(1.06, 1.70)	0.84	(0.48, 1.48)
Deprivation 50-89%	1.96	(1.40, 2.73)	1.11	(0.95, 1.30)	0.85	(0.58, 1.26)
Deprivation >90%	*Reference*		*Reference*		*Reference*	
Non-ARI admissions	1.00	(1.00, 1.00)	1.00	(1.00, 1.00)	1.00	(1.00, 1.00)
**Total ARI**						
Year[a]	0.99	(0.98, 1.00)	1.05	(1.04,1.06)	1.03	(1.02, 1.04)
Male	*Reference*		*Reference*		*Reference*	

For peer review only - http://bmjopen.bmj.com/site/about/guidelines.xhtml

Female	0.68	(0.65, 0.72)	0.69	(0.64, 0.73)	0.71	(0.65, 0.77)
Deprivation 0-10%	3.85	(3.50, 4.21)	1.87	(1.70, 2.06)	1.25	(1.14, 1.37)
Deprivation 10-49%	2.22	(1.95, 2.54)	1.41	(1.07, 1.85)	1.25	(0.88, 1.77)
Deprivation 50-89%	1.42	(1.26, 1.60)	1.14	(0.96, 1.36)	1.08	(0.85, 1.36)
Deprivation >90%	*Reference*		*Reference*		*Reference*	
Non-ARI admissions	1.00	(1.00, 1.00)	1.00	(1.00, 1.00)	1.00	(1.00, 1.00)

IRR, Incidence Rate Ratio

[a]Year included as a linear term

For peer review only

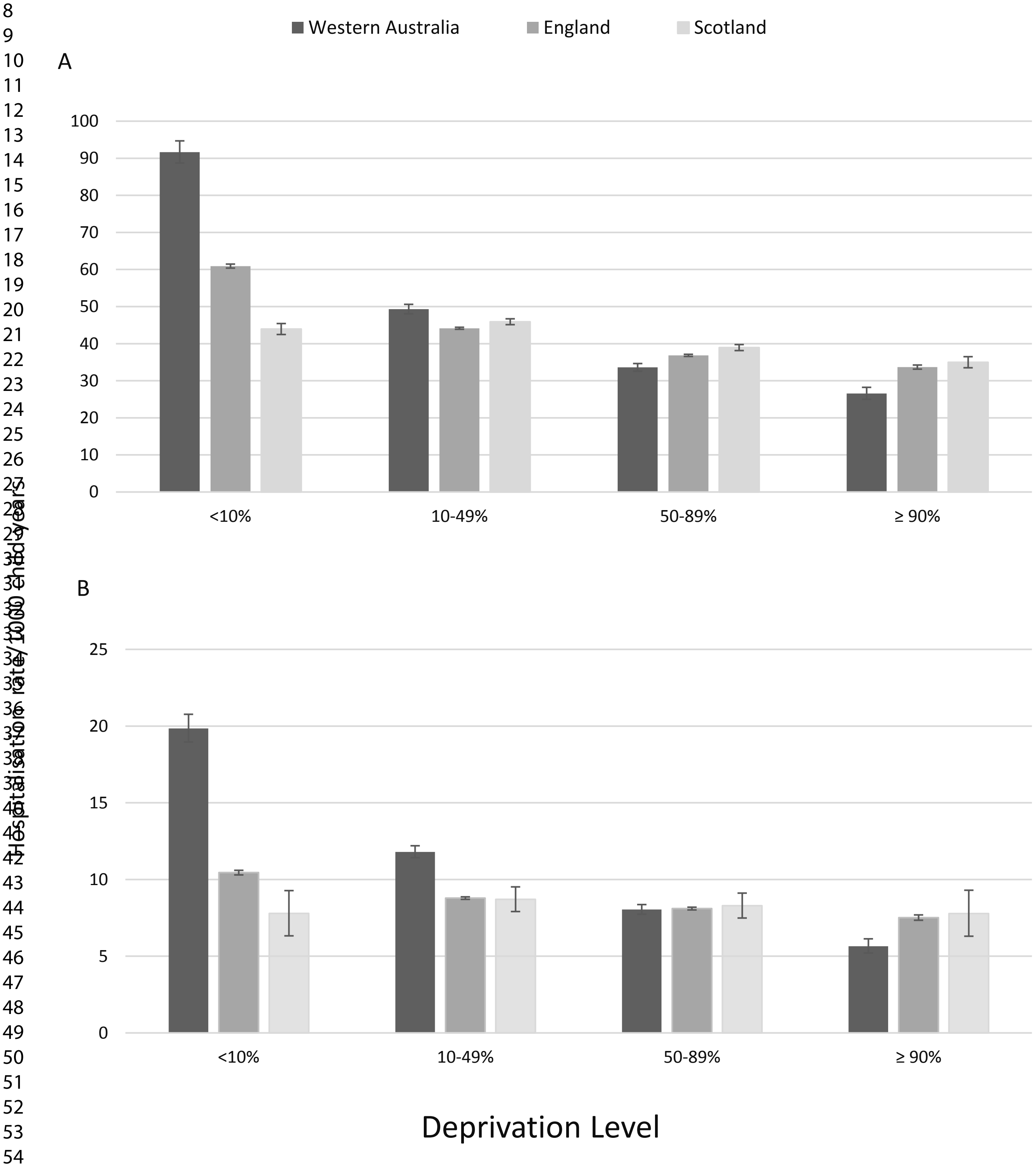
Western Australia
England
Scotland
A
100
90
80
70
60
50
40
30
20
10
0
<10%
10-49%
50-89%
≥ 90%
B
25
20
15
10
5
0
<10%
10-49%
50-89%
≥ 90%
Hospitalisation rate/1000 child years
Deprivation Level

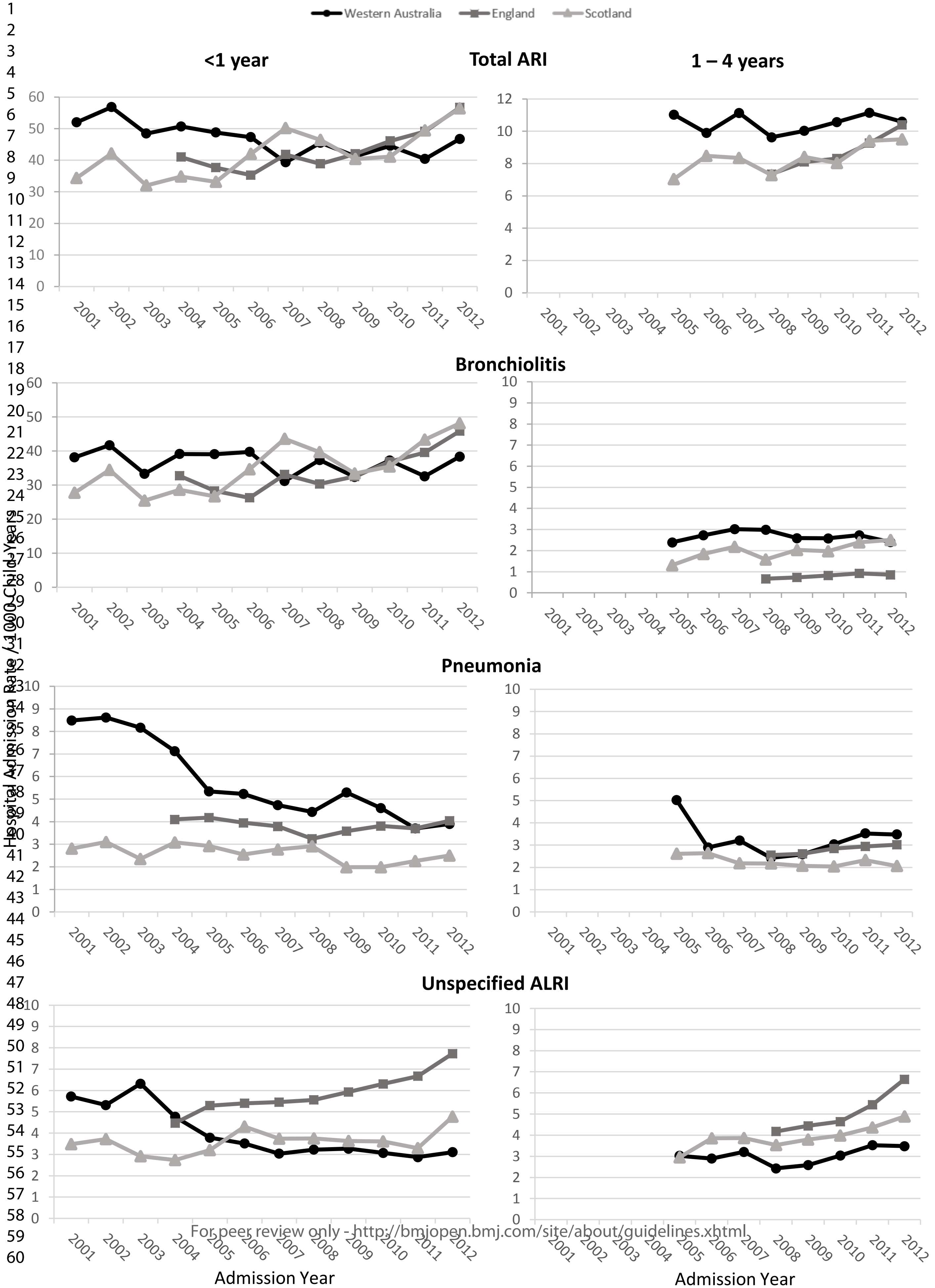
Western Australia
England
Scotland
<1 year
Total ARI
1 – 4 years
Bronchiolitis
Pneumonia
Unspecified ALRI
Hospital Admission Rate / 1000 Child-Years
Admission Year
Admission Year

Supplementary Table 1: Cohort characteristics by jurisdiction

Characteristic		Western Australia		England		Scotland	
		n	(%)	n	(%)	n	(%)
Sex[a]	Male	173,081	(51.2)	3,044,931	(51.3)	359,159	(51.3)
	Female	164,828	(48.8)	2,894,078	(48.7)	340,430	(48.7)
Year of birth	2000	22,551	(6.7)	-		51,479	(7.4)
	2001	22,461	(6.7)	-		50,295	(7.2)
	2002	22,412	(6.7)	-		49,425	(7.1)
	2003	22,345	(6.6)	535,724	(9.0)	50,450	(7.2)
	2004	23,361	(6.9)	551,939	(9.3)	52,417	(7.5)
	2005	24,776	(7.3)	560,894	(9.4)	52,415	(7.5)
	2006	26,627	(7.9)	579,220	(9.8)	53,830	(7.7)
	2007	26,943	(8.0)	586,526	(9.9)	55,765	(8.0)
	2008	28,216	(8.4)	614,471	(10.4)	57,481	(8.2)
	2009	28,588	(8.5)	611,427	(10.3)	57,007	(8.2)
	2010	28,565	(8.5)	629,778	(10.7)	56,586	(8.1)
	2011	29,535	(8.7)	632,306	(10.7)	56,510	(8.1)
	2012	31,529	(9.3)	636,724	(10.7)	55,929	(8.0)
Deprivation[b]	<10% (most deprived)	40,936	(12.1)	885,891	(14.9)	85,987	(12.3)
	10-49%	122,309	(36.2)	2,635,562	(44.4)	295,372	(42.2)
	50-89%	131,232	(38.8)	1,957,789	(33.0)	252,407	(36.1)
	≥90% (least deprived)	43,432	(12.9)	459,767	(7.6)	65,823	(9.4)
**TOTAL**		**337,909**	**(100.0)**	**5,939,009**	**(100.0)**	**699,589**	**(100.0)**

[a] Sex was missing was 7205 children (England) with no missing data from Western Australia or Scotland.

[b] Deprivation scores were missing for 18,920 children (Western Australia), 146,588 (England) and 827 (Scotland).

For peer review only

Supplementary Table 2: Hospitalisation rate for ARI by diagnostic category by primary diagnosis only (PDx) and primary diagnosis plus all additional diagnoses (Any Dx) in infants aged <1 year and children aged 1-4 years in Western Australia, England and Scotland

	Western Australia			England			Scotland		
	Rate[a]		IRR (95% CI)	Rate[a]		IRR (95% CI)	Rate[a]		IRR (95% CI)
**ARI diagnosis**	**Any Dx**	**PDx**		**Any Dx**	**PDx**		**Any Dx**	**PDx**	
**<1 year[b]**									
Whooping cough	0.8	0.7	1.2 (1.0, 1.4)	0.5	0.5	1.1 (1.0, 1.2)	0.6	0.6	1.1 (0.9, 1.2)
Pneumonia	5.6	4.1	1.4 (1.3, 1.5)	3.8	2.9	1.3 (1.3, 1.3)	2.6	1.9	1.3 (1.2, 1.4)
Bronchiolitis	36.6	34.4	1.1 (1.0, 1.1)	34.2	32.2	1.1 (1.1, 1.1)	35.4	34.3	1.0 (1.0, 1.1)
Influenza	1.6	1.3	1.3 (1.1, 1.4)	0.4	0.3	1.4 (1.3, 1.5)	0.9	0.7	1.3 (1.2, 1.5)
Unspecified ARI	3.9	2.6	1.5 (1.4, 1.6)	5.9	4.6	1.3 (1.3, 1.3)	3.6	2.8	1.3 (1.2, 1.4)
Bronchitis	0.7	0.5	1.3 (1.1, 1.6)	0.5	0.4	1.2 (1.1, 1.3)	0.5	0.4	1.2 (1.1, 1.5)
Total ARI	46.4	43.7	1.1 (1.0, 1.1)	43.4	40.1	1.1 (1.1, 1.1)	42.1	40.7	1.0 (1.0, 1.1)
**1-4 years[c]**									
Whooping cough	0.05	0.04	1.1 (0.7, 1.9)	0.009	0.008	1.1 (0.9, 1.5)	0.02	0.01	1.1 (0.7, 2.0)
Pneumonia	4.3	3.7	1.2 (1.1, 1.2)	2.8	2.5	1.1 (1.1, 1.1)	2.3	2.1	1.1 (1.1, 1.1)
Bronchiolitis	2.7	2.3	1.2 (1.1, 1.2)	0.8	0.7	1.1 (1.1, 1.2)	2.0	1.9	1.1 (1.0, 1.1)
Influenza	0.6	0.4	1.3 (1.1, 1.4)	0.2	0.2	1.3 (1.2, 1.4)	0.3	0.2	1.2 (1.1, 1.4)
Unspecified ARI	3.0	2.2	1.4 (1.3, 1.5)	5.1	4.2	1.2 (1.2, 1.2)	3.9	3.3	1.2 (1.1, 1.2)
Bronchitis	0.3	0.2	1.4 (1.2, 1.7)	0.1	0.1	1.2 (1.1, 1.3)	0.1	0.1	1.3 (0.0, 1.6)
Total ARI	10.5	9.0	1.2 (1.1, 1.2)	8.7	7.6	1.1 (1.1, 1.1)	8.4	7.6	1.1 (0.0, 1.1)

IRR, incidence rate ratio

For peer review only

[a]Rate is per 1000/child-years

[b]2001-2012 for Western Australia and Scotland; 2004-2012 for England

[c]2005-2012 for Western Australia and Scotland; 2008-2012 for England

For peer review only

Temporal trends and socio-economic differences in acute respiratory infection hospitalisations in children: an inter-country comparison of birth cohort studies in Western Australia, England and Scotland

Journal:	*BMJ Open*
Manuscript ID	bmjopen-2018-028710.R1
Article Type:	Research
Date Submitted by the Author:	26-Feb-2019
Complete List of Authors:	Moore, Hannah; Wesfarmers Centre of Vaccines and Infectious Diseases, Telethon Kids Institute, The University of Western Australia,<br>de Klerk, Nicholas; Telethon Kids Institute, Wesfarmers Centre of Vaccines and Infectious Diseases<br>Blyth, Christopher C.; Division of Paediatrics, School of Medicine, The University of Western Australia; PathWest Laboratory Medicine WA, Perth Children's Hospital<br>Gilbert, Ruth; University College London, Institute of Child Health<br>Fathima, Parveen; Wesfarmers Centre of Vaccines and Infectious Diseases, Telethon Kids Institute, The University of Western Australia<br>Zylbersztejn, Ania; University College London Great Ormond Street Institute of Child Health , Population, policy and practice<br>Verfürden, Maximiliane ; University College London, Great Ormond Street Institute of Child Health<br>Hardelid, Pia; UCL Institute of Child Health, Centre for Paediatric Epidemiology and Biostatistics
**Primary Subject Heading**:	Epidemiology
Secondary Subject Heading:	Paediatrics, Infectious diseases
Keywords:	Respiratory infections < THORACIC MEDICINE, socio-economic disparity, infant, hospitalisation, record linkage

Temporal trends and socio-economic differences in acute respiratory infection hospitalisations in children: an inter-country comparison of birth cohort studies in Western Australia, England and Scotland

Hannah C Moore[1], Nicholas de Klerk[1], Christopher C Blyth[1, 2, 3, 4], Ruth Gilbert[5], Parveen Fathima[1], Ania Zylbersztejn[5], Maximiliane Verfürden[5], Pia Hardelid[5]

[1]Wesfarmers Centre of Vaccines & Infectious Diseases, Telethon Kids Institute, The University of Western Australia, Perth, Australia

[2]School of Medicine, The University of Western Australia, Perth, Australia

[3]Department of Infectious Diseases, Princess Margaret Hospital for Children, Perth, Australia

[4]PathWest Laboratory Medicine WA, QE11 Medical Centre, Perth, Australia

[5]University College London Great Ormond Street Institute of Child Health, London, United Kingdom

Author for Correspondence:

Hannah C Moore, Wesfarmers Centre of Vaccines & Infectious Diseases, Telethon Kids Institute, The University of Western Australia. PO Box 855, West Perth, Western Australia 6872

Email: hannah.moore@telethonkids.org.au

Phone: + 61 8 6319 1427 / +61 409 100 007

For peer review only - http://bmjopen.bmj.com/site/about/guidelines.xhtml

ABSTRACT

Objectives: Acute respiratory infections (ARI) are a global cause of childhood morbidity. We compared temporal trends and socioeconomic disparities for ARI hospitalisations in young children across Western Australia, England and Scotland.

Design: Retrospective population-based cohort studies using linked birth, death and hospitalisation data.

Setting and Participants: Population birth cohorts spanning 2000-2012 (Western Australia and Scotland) and 2003-2012 (England).

Outcome Measures: ARI hospitalisations in infants (<12 months) and children (1-4 years) were identified through ICD-10 diagnosis codes. We calculated admission rates per 1000 child-years by diagnosis and jurisdiction- specific socio-economic deprivation and used negative binomial regression to assess temporal trends.

Results: The overall infant ARI admission rate was 44.3/1000 child-years in Western Australia, 40.7/1000 in Scotland and 40.1/1000 in England. Equivalent rates in children aged 1-4 years were 9.0, 7.6, and 7.6. Bronchiolitis was the most common diagnosis. Compared with the least socio-economically deprived, those most deprived had higher ARI hospitalisation risk (incidence rate ratio 3.9 [95% confidence interval 3.5, 4.2] for Western Australia; 1.9 [1.7, 2.1] for England; 1.3 [1.1, 1.4] for Scotland). ARI admissions in infants were stable in Western Australia but increased annually in England (5%) and Scotland (3%) after adjusting for non-ARI admissions, sex, and deprivation.

Conclusions: Admissions for ARI were higher in Western Australia and displayed greater socio-economic disparities than England and Scotland, where ARI rates are increasing. Prevention programs focusing on disadvantaged populations in all three countries are likely to translate into real improvements in the burden of ARI in children.

For peer review only - http://bmjopen.bmj.com/site/about/guidelines.xhtml

For peer review only

Keywords (3-10)

Acute respiratory infections; hospitalisation; socio-economic disparity; international comparison; infant; population; record linkage

ARTICLE SUMMARY

Strengths and limitations of this study

- We used population-level data from three countries to assess hospitalisation rates and changes over time for acute respiratory infections in children
- Analysis protocols and diagnosis coding was standardised across each country
- Hospitalisation rates for acute respiratory infections were described according to level of socio-economic deprivation
- To control for changing admission thresholds within each country, we adjusted our models for all non-acute respiratory infection admissions
- A limitation of this study is the different measures of socio-economic deprivation available across the three countries

For peer review only - http://bmjopen.bmj.com/site/about/guidelines.xhtml

BACKGROUND

Acute respiratory infections (ARI) including bronchiolitis, pneumonia and influenza are a major cause of hospitalisation in children worldwide, responsible for approximately 12 million annual episodes in children under 5 years of age[1, 2]. In England, the hospital admission rate for ARI increased by 40% from 1999-2010 among children aged less than 15 years[3] and bronchiolitis was the most common reason for unplanned admissions in infants from 2010-2013.[4] While hospitalisations for ARI doubled from 1992-2000 in Western Australia,[5] they since stabilised 2000-2005.[6] Vaccination programmes including influenza, pertussis and pneumococcal disease have been implemented in North America, Europe and Australia, but the majority of ARI hospitalisations in high income countries are now caused by non-vaccine preventable viruses including Respiratory Syncytial Virus (RSV), Parainfluenza virus and Human Metapneumovirus.[7]

ARI hospitalisations are more common among children from poorer socio-economic backgrounds.[8, 9] In addition to access to inadequate health care, risk factors for developing severe symptoms of ARIs, including prematurity, low birth weight, congenital anomalies, exposure to environmental tobacco smoke, damp and mould, and household overcrowding are all more common among children growing up in more deprived families in both high and low income settings.[10, 11] Understanding the impact of socio-economic disparities on ARI hospitalisations among children (both over time and between countries) can provide an estimate of the preventable proportion of ARI. Linkage of administrative health datasets provides a platform to investigate these trends in populations over many years. Additionally, the availability of comparable hospital admission datasets with similar coding systems using International Classification of Diseases, 10th edition (ICD-10) diagnosis codes allows comparison of hospitalisation rates among children for ARI according to deprivation level.

For peer review only - http://bmjopen.bmj.com/site/about/guidelines.xhtml

Using record linkage resources within Western Australia, England and Scotland, we conducted a comparative analysis of the three jurisdictions to investigate the hospitalisation rates for ARI in children aged less than 5 years. All three jurisdictions have publicly funded health care with free access to primary and public hospital care. Each jurisdiction has established childhood vaccination programs targeting acute respiratory infections. This includes diphtheria, tetanus, pertussis, *Haemophilus influenzae* type B (3 dose infant schedule), pneumococcal disease (2 + 1 schedule) and recently, seasonal influenza. Excluding influenza, vaccination coverage at age 12 months is >90% for all 3 jurisdictions.[12, 13] Our aim was to compare population-based hospitalisation rates by ARI diagnosis, age and level of socio-economic deprivation, and assess how ARI hospitalisation rates have changed over time.

METHODS

Data Sources and Study Populations

We conducted separate population-based birth cohort studies using administrative data from Western Australia, England and Scotland. Western Australia covers the western third of Australia, an area of 2.5 million square kilometres with a population of nearly 2.6 million,[14] 3.6% of whom identify as being Aboriginal and/or Torres Strait Islander (herein referred to as Aboriginal).[15] Births were identified from the Midwives' Notification System and Birth Register, deaths were identified from the Death Register and hospitalisations were recorded in the Hospital Morbidity Database Collection that provides full coverage of all hospital separations (hereafter referred to as hospitalisations). In the absence of a unique person identifier in Australia, extracted data were probabilistically linked by the Western Australian Data Linkage Branch using a series of demographic identifiers using an established best practice protocol.[16, 17] Aboriginal status was derived using a

For peer review only - http://bmjopen.bmj.com/site/about/guidelines.xhtml

validated algorithm using Aboriginal identification information across all available records.[18] England has a population of 53.9 million.[19] The birth cohort was established by linking hospital birth and delivery records from the Hospital Episode Statistics (HES) database.[20] Hospitalisations and deaths were identified via linkage to mortality registration data from the Office for National Statistics.[21] Data linkage in England was carried out by NHS Digital, using a deterministic algorithm based on the NHS number (a unique patient identifier in the English NHS), postcode, date of birth, sex and local hospital numbers. Scotland has a population of 5.3 million.[19] The Scottish birth cohort was developed through linking data from birth registration and maternity databases[22, 23]. Hospitalisations and deaths were identified via linkage to the Scottish Morbidity Record 01 (SMR-01) and mortality records using deterministic linkage carried out by the electronic Data Research and Innovation Service (eDRIS) based on the Community Health Index number, a unique identifier recorded on all births and subsequent encounters within the Scottish NHS.

The datasets represented 99.9% of all births in Western Australia, 97.5% in England[24] and 100% in Scotland with full coverage of inpatient and day admissions. Our study population comprised of singleton births in Western Australia and Scotland 2000-2012 and England 2003-2012. Multiple births were excluded due to a higher likelihood of linkage error. Children were followed from birth until their fifth birthday, date of death, or 30 June 2013 (the end of follow-up) or (Scotland only) date of emigration, whichever occurred first.

Outcome Measures

Our outcome measure was an ARI emergency hospitalisation for children in their first 5 years of life. All inter-hospital transfers were collapsed into a single admission. We identified hospitalisations for ARI using a selection of ICD-10 diagnosis codes (ICD-10-AM for Western Australia).[25]

For peer review only - http://bmjopen.bmj.com/site/about/guidelines.xhtml

Hospitalisation data for each jurisdiction provided a principal diagnosis code and up to 20 secondary diagnosis codes in Western Australia, 19 in England and 5 in Scotland. We identified ARI hospitalisations using the principal diagnosis code and all the available additional diagnosis codes as six diagnostic groups: whooping cough (A37), influenza (J09-J11), pneumonia (J12-J18, B59, B05.2, B37.1, B01.2), bronchitis (J20, J40), bronchiolitis (J21) and unspecified acute lower respiratory infection.(J22) Consistent with our previous Western Australian work,[6] ARI hospitalisations within 14 days of a previous ARI hospitalisation were classified as a single infection episode. In such cases we applied a hierarchical diagnosis algorithm[6] within the readmission set in order to code an overall principal diagnosis. This algorithm ranked diagnoses in order of disease severity: whooping cough, pneumonia, bronchiolitis, influenza, unspecified ALRI and bronchitis. Children with missing data on sex or deprivation were excluded from the analyses.

Exposure Measures

We assessed hospitalisations for ARI in infants aged less than 12 months and young children aged 1-4 years at time of admission. Other exposure measures of interest were sex, level of socio-economic deprivation and admission year. In Western Australia, socio-economic deprivation was measured through the Index of Relative Disadvantage (IRSAD), one of the four Socio-Economic Indexes for Areas (SEIFA) derived by the Australian Bureau of Statistics.[26] The IRSAD score is derived from 17 different variables including low income, internet connection, unemployment and education.[26] Scores were grouped into Collectors District, the smallest unit for population-based analyses which, on average, consist of approximately 200 dwellings. For England, socio-economic deprivation was measured through the Index of Multiple Deprivation (IMD), based on seven domains of deprivation including income, employment, education, crime, barriers to housing and living environment.[27] IMD scores are measured at Lower Super Output Area Level, covering an average of 1200-1500 households. For Scotland, deprivations scores were based on the Carstairs Index, based on four

For peer review only - http://bmjopen.bmj.com/site/about/guidelines.xhtml

variables including car ownership, male unemployment, overcrowding and low occupational social class. The Carstairs Index is measured at postcode sector level, which contains an average of 5000 people[28] In all jurisdictions socio-economic deprivation scores were based on mother's residential address at time of her child's delivery and were grouped into four levels based on a country level ranking with the lowest scores representing the most socio-economically deprived.

Statistical Analysis

Consistent methodology was applied to the assembled datasets in the three jurisdictions. We calculated hospitalisation rates per 1000 child-years at risk for each diagnostic grouping of ARI (as principal diagnosis). To assess the impact of including additional diagnosis codes, we compared hospitalisation rates derived using the principal diagnosis code only with rates derived from using the principal plus all additional diagnosis codes (any diagnosis). We used any diagnosis to assess ARI rates by socio-economic deprivation and year of admission. We present age-specific hospitalisation rates with 95% confidence intervals (CI) and where appropriate, rates were compared using incidence rate ratios (IRRs) with 95% CIs. To assess temporal trends, we plotted annual hospitalisation rates in the two age groups for each jurisdiction by admission year for all ARIs and bronchiolitis, pneumonia and unspecified ALRI's. We also used negative binomial regression models to assess linear temporal trends in infant hospitalisations from 2001-2012 (Western Australia and Scotland) and 2004-2012 (England). Year of admission was included as a linear term in the models, and the natural logarithm of child-years at risk was included as an offset in the models. Trends over time in ARI admission rates were assumed to be statistically significant if the Wald test *p*-value for the coefficient for the linear year term was <0.05. Models were adjusted for sex and the 4-level socioeconomic indicator and we present IRR's with 95% CI's. In order to control for overall trends in hospitalisation we also adjusted the models for the number of all non-ARI emergency admissions.[29] All data analyses were conducted within each jurisdiction in Stata version 14.0.[30]

For peer review only - http://bmjopen.bmj.com/site/about/guidelines.xhtml

Public and Patient Involvement

A community reference group located in Western Australia was consulted during the conduct of this study. No individual patients were involved.

RESULTS

A total of 337,909 (Western Australia), 5,939,009 (England) and 699,590 (Scotland) births were included in the study (Supplementary Table 1). There were 14,480 infant hospitalisations for ARI as a principal diagnosis in Western Australia, 217,985 for England and 26,103 for Scotland giving overall infant hospitalisation rates of 44.3/1000 child-years for Western Australia, 40.7/1000 for Scotland and 40.1/1000 for England. In all jurisdictions, bronchiolitis had the highest hospitalisation rates accounting for 79% of ARI admissions in infants in Western Australia, 79% in England, and 84% in Scotland (Table 1). ARI hospitalisation rates in infants were higher in Western Australia compared with England and Scotland across all ARI diagnoses, most notably for pneumonia, where rates were 1.4-2.2 times higher compared to England and Scotland. The only exception was for unspecified ALRI where the hospitalisation rate in infants were 70% higher in England than in Scotland and Western Australia. ARI hospitalisation rates in children aged 1-4 years were 19% higher in Western Australia compared with England and Scotland (Table 1). The most common ARI principal diagnosis among children aged 1-4 years was pneumonia in Western Australia (42%) and unspecified ALRI in England (54.6%) and Scotland (43.9%). Consequently, hospitalisation rates for pneumonia in Western Australian children aged 1-4 years were 1.5-1.8 times higher than England and Scotland.

For peer review only - http://bmjopen.bmj.com/site/about/guidelines.xhtml

When ARI hospitalisations were identified based on any diagnosis compared with principal diagnosis only, the difference in hospitalisation rates varied across diagnoses with the most notable difference for unspecified ALRI in Western Australia where rates were 1.5 (95% CI: 1.4, 1.6) times higher in infants when using any diagnosis compared with principal diagnosis only (Supplementary Table 2).

ARI hospitalisation rates were higher for children from the most socio-economically deprived areas. The association with deprivation was greatest in Western Australia and more marked in infants compared to young children aged 1-4 years (Figure 1). The relative difference in ARI hospitalisation rates between the most and least deprived infants was 3.5 (95% CI: 3.2, 3.7) in Western Australia; 1.8 for England and 1.3 for Scotland with similar patterns in children aged 1-4 years (Figure 1). In multivariable models, level of socio-economic deprivation was significantly associated with all ARI categories in all infants but most notably in Western Australia, and in particular, pneumonia (IRR 6.9, 95% CI: 5.6, 8.6) and unspecified ALRI (IRR 8.9, 95% CI: 6.7, 11.8; Table 2).

Overall, ARI hospitalisation rates have increased in England and Scotland, but declined (infants) or remained stable (children aged 1-4 years) in Western Australia (Figure 2). After adjusting for sex, deprivation and non-ARI emergency hospitalisations, the ARI hospitalisation rate among infants increased by 5% per year in England (IRR 1.05, 95% CI: 1.04, 1.07) and by 3% per year (IRR 1.03, 95% CI: 1.02, 1.04) in Scotland with no statistically significant trend in Western Australia (IRR 0.99, 95% CI: 0.98, 1.00; Table 2, Figure 2). Similar results were seen for bronchiolitis admissions in infants.

Diverging trends were seen with pneumonia and unspecified ALRI across the three jurisdictions with pneumonia hospitalisation rates in infants declining in Western Australia from 9.0/1000 in 2002 to 3.9/1000 in 2012 while rates remained steady around 3-4/1000 in England and 2-3/1000 in Scotland

For peer review only - http://bmjopen.bmj.com/site/about/guidelines.xhtml

(Figure 2). After adjusting for sex, socio-economic deprivation and non-ARI admissions, the annual decline in pneumonia hospitalisations was 6% in Western Australia (IRR 0.94, 95%CI: 0.93, 0.96), 2% in England and 3% in Scotland (Table 2). Unspecified ALRI declined in Western Australia annually by 5% but increased by 6% and 2% annually in England and Scotland (Table 2).

DISCUSSION

ARI, particularly bronchiolitis, continues to be an important cause of infant and childhood hospitalisation. The availability of linked administrative data in three economically similar jurisdictions with publicly funded healthcare systems afforded us the opportunity to compare ARI hospitalisation rates in children. Overall, admission rates were highest in Western Australia and decreasing or remaining stable but increasing in England and Scotland. The relative differences in ARI admission rates between children from the most socioeconomically deprived areas to the least deprived areas were largest in Western Australia.

The interpretation of hospitalisation trends across countries is complex. We have found higher rates of ARI admissions in Western Australia compared with England and Scotland which could mean a higher incidence in ARI, a higher risk of developing more severe symptoms, or differences in diagnostic coding or hospital admission thresholds. A recent study comparing admission rates between England and Ontario finding substantially higher rates in England was partly explained by differing admission thresholds from differential waiting practices and policies in emergency departments.[4] Comparisons of asthma admissions from national hospital data in Finland and Sweden noted diverging trends citing differences in national coding guidelines and subsequent altered admission thresholds.[31] In an attempt to control for changing admissions thresholds over time within each jurisdiction, we adjusted our multivariable models for the overall trend in non-ARI

For peer review only - http://bmjopen.bmj.com/site/about/guidelines.xhtml

emergency hospital use. However we could not adjust for differing thresholds between countries. Emergency hospitalisations are increasing at a faster rate in England compared to other parts of the United Kingdom[32] and our data here suggests that hospitalisations due to unspecified ALRI and bronchiolitis in England are contributing to that increase. It is also possible that diverging trends are a result of diagnostic shifts in that for the same clinical presentations, a diagnosis of unspecified ALRI is given in England while other non-specific codes (including codes we have not assessed) are given in Western Australia and Scotland. The use of additional diagnosis codes for ARI seemed more frequent in Western Australia compared with England and Scotland and should be taken into consideration for future comparative studies using ICD diagnosis codes.

Hospitalisation rates for ARI were significantly associated with level of socio-economic deprivation, consistent with an earlier analysis in England.[33] This association was strongest in Western Australia with IRRs for those in the most deprived level in the order of 3.9 for all ARIs, up to 8.9 for unspecified ALRI. There appeared a linear relationship with level of deprivation and rates of ARI in Western Australia while rates in all levels (bar the most deprived) not differing in England and Scotland. Western Australian data were inclusive of Aboriginal children, an Indigenous population with higher levels of socio-economic disadvantage[34] compared to their non-Aboriginal peers and a significantly higher burden of pneumonia worldwide,[6, 35, 36] despite reductions in the 2000's and further reductions seen in our results here, most likely due to the positive impact of pneumococcal vaccination.[6, 37] This most likely explains the higher rates of pneumonia seen in Western Australia compared with England and Scotland. We have previously reported that hospitalisation rates for all acute respiratory infections are 5 to 7 times higher in young Aboriginal children compared with non-Aboriginal children.[9] Aboriginal children also suffer a disproportionate burden of RSV,[38] the major cause of bronchiolitis which could explain the higher bronchiolitis rates in Western Australia than in England and Scotland. However level of socio-economic deprivation has been associated

For peer review only - http://bmjopen.bmj.com/site/about/guidelines.xhtml

with hospitalisations for respiratory infections in both Aboriginal and non-Aboriginal children[9] so the contribution of Aboriginal children alone cannot explain the higher socio-economic disparities seen here. Indeed, when Aboriginal children were removed from the analysis, the socio-economic disparities remained, although slightly lessened, and were still higher than England and Scotland (e.g. the IRR for most deprived children for all ARI reduced from 3.9 to 2.1 and for unspecified ALRI reduced from 8.9 to 2.9 (data not shown)). Respiratory infections continue to be a source of health inequalities among disadvantaged children worldwide. Geographical remoteness is more of an issue in Western Australia due to its sheer geographical size in comparison to England and Scotland. The lack of adequate primary care in rural and remote Australia[39] which is often coupled with lower socio-economic levels could be driving higher hospitalisation rates. Nevertheless, these important findings highlight the need for targeted prevention programs such as smoking cessation, improved housing and timely vaccination for key respiratory pathogens for the most disadvantaged populations in all three jurisdictions.

Since 2013, the United Kingdom had been rolling out a universal seasonal influenza vaccination program for children aged 2 years to 16 years and from 2018, all Australian states and territories offer free seasonal influenza vaccine to children aged between 6 months and 5 years. Our study period was prior to this time. Relative to other ARI diagnoses, recorded influenza hospitalisation rates are low. Assessing the impact of the universal childhood vaccination program for influenza is likely to be challenging without linking national-level birth cohorts to infection surveillance and vaccination data. This has already been implemented in Scotland.[40] There is also renewed interest in preventing morbidity due to RSV with vaccination.[41] Understanding the baseline hospitalisation rates for RSV-bronchiolitis and pneumonia prior to when vaccination is available is critical to aid in implementation and for its ongoing evaluation post implementation.

For peer review only - http://bmjopen.bmj.com/site/about/guidelines.xhtml

We conducted our analysis on near total population birth cohorts in each jurisdiction and thus our outcome measures have narrow confidence intervals and minimum selection bias. An additional strength of the population-based cohort design is standardisation of analysis protocols and the provision of large numbers allowing us to assess temporal trends and associations with less common infections. The hospital morbidity database systems used in all three jurisdictions have the same population coverage of all inpatient admissions and day surgeries further adding to the validity of our estimates. Although Western Australia is a state within Australia and we have made comparisons to country wide data for England and Scotland, the rate of cross-border hospitalisation from Western Australia to other Australian states is very low.[42]

However, our study does have some limitations. The socio-economic deprivation scores used were jurisdiction specific and included different items to represent disadvantage. In addition, area-level socio-economic deprivation was only measured at birth. Therefore, the observed association between area-level socio-economic deprivation and the rate of ARI admissions may be subject to increasing measurement error as the child's age increases. How socio-economic deprivation is associated with morbidity due to ARI at the primary care level is unknown but perhaps likely to aid in explaining disparities in socio-economic deprivation that we have seen here. While primary care data is more readily available in England and Scotland, limited data with adequate diagnostic information is available for population-based studies in Western Australia. As previously alluded to, there also may be differences in admission thresholds across the three jurisdictions that may explain some higher admission rates across countries. A comparison of emergency department presentations in conjunction with hospitalisations for ARI could be useful here, although diagnostic information from emergency department data is limited[43] and no individual level data on emergency department visits exist in Scotland. Additionally, through our experience of linking routine laboratory data to hospital data in Western Australia, we are aware of unspecific ICD codes

For peer review only - http://bmjopen.bmj.com/site/about/guidelines.xhtml

that are associated with detections of respiratory viral pathogens.[44] We did not include such ICD codes (e.g. viral infection of unspecified site "B34") in this analysis. However we would not expect the potential exclusion of ARI hospitalisations to alter the direction of our results in terms of association with socio-economic deprivation.

CONCLUSIONS

Population-based administrative data from economically similar developed countries provides a powerful tool to conduct international comparative studies that can compare and contrast the epidemiology of, and healthcare responses to, respiratory infections. Western Australia experiences higher admissions in children for ARI and a greater disparity in rates according to level of socio-economic deprivation. Rates are overall slightly lower in England and Scotland but are increasing, particularly in England. These findings suggest that prevention programs focusing on disadvantaged populations in all three countries are likely to translate into real improvements in the burden of ARI in children. We are planning to use these administrative data to assess effectiveness of interventions (such as vaccination) and how this may affect disparities in ARI admissions rates according to socio-economic deprivation.

Acknowledgements

We would like to acknowledge the Western Australian Data Custodians, and particularly Alexandra Merchant and Mikhalina Dombrovskaya from the Western Australian Data Linkage Branch, for their assistance and support in collating the data for Western Australia. Source data from England can be accessed by researchers applying to the Health and Social Care Information Centre for England. Copyright © 2017, Re-used with the permission of NHS Digital. All rights reserved. This research

For peer review only

benefits from and contributes to the NIHR Children and Families Policy Research Unit, but was not commissioned by the NIHR Policy Research Programme.

List of Abbreviations

ALRI: Acute lower respiratory infection; ARI: Acute respiratory infection; CI: Confidence interval; HES: Hospital Episode Statistics; ICD: International Classification of Diseases; IMD: Index of Multiple Deprivation; IRR: Incidence rate ratio; IRSAD: Index of Relative Disadvantage; NHS: National Health Service; RSV: Respiratory syncytial virus; SEIFA: Socio-Economic Index for Australia

Competing interests

The authors do not have any commercial or other association that might pose a conflict of interest.

Funding

This work was supported by a National Health and Medical Research Council Project Grant [1045668], a University of Western Australia Research Collaboration Award [to HCM] and a Wesfarmers Centre of Vaccines & Infectious Diseases Seed Grant [to HCM, CCB, PH]. CCB and HCM are supported by National Health and Medical Research Council Fellowships [1034254 to HCM and 1111596 to CCB]. PH was funded by a National Institute for Health Research postdoctoral Fellowship, reference number PDF-2013–06–004. The views expressed in this publication are those of the author(s) and not necessarily those of the NHS, the National Institute for Health Research or the Department of Health. AZ's PhD studentship is funded by awards to establish the Farr Institute of Health Informatics Research, London, from the Medical Research Council, Arthritis Research UK, British Heart Foundation, Cancer Research UK, Chief Scientist Office, Economic and Social Research

For peer review only - http://bmjopen.bmj.com/site/about/guidelines.xhtml

Council, Engineering and Physical Sciences Research Council, National Institute for Health Research, National Institute for Social Care and Health Research, and Wellcome Trust (grant MR/K006584/1). AZ is also supported by the Administrative Data Research Centre for England (funded by the Economic and Social Research Council). MV's PhD studentship is funded by the UBEL DTP of the Economic and Social Research Council. MV was also supported by the Policy Research Unit in the Health of Children, Young People and Families (reference 109/00017), which is funded by the Department of Health Policy Research Programme at UCL. This is an independent report commissioned and funded by the Department of Health. The views expressed are not necessarily those of the Department.

For peer review only

Ethics approval and consent to participate

Approval to use the Western Australian data was granted by the Western Australian Department of Health Human Research Ethics Committee, the Western Australian Aboriginal Health Ethics Committee and the Western Australian Data Linkage Branch. We have a data sharing agreement with National Health Service (NHS) Digital to use a de-identified extract of Hospital Episode Statistics for research into children's use of secondary care services; therefore, we did not require ethical approval to use English datasets. For Scotland, approvals were obtained from the Public Benefit and Privacy Panel for Health and Social Care, reference number 1516-0405.

Consent for publication

Not applicable.

For peer review only - http://bmjopen.bmj.com/site/about/guidelines.xhtml

Data Sharing Statement

We cannot share the individual-level data used for this study under our agreements with the data providers. The datasets analysed during the current study can be applied for from the Western Australian Data Linkage System (Western Australia; http://www.datalinkage-wa.org.au/), NHS Digital; (England; http://content.digital.nhs.uk) and the electronic Data Research and Innovation Service (Scotland; http://www.isdscotland.org/Products-and-Services/eDRIS/). Derived data from these datasets for each jurisdiction are within the paper. No additional data are available.

For peer review only

Authors' contributions

HCM, CCB and PH conceived the study design. PF assisted with data cleaning and coding in Western Australia, AZ and MV assisted with data extraction for England and Scotland. Statistical analysis was conducted by HCM (Western Australia) and PH (England and Scotland) with expert advice from NdK with critical revisions for intellectual content from CCB and RG. HCM drafted the first manuscript with PH. All authors read and approved the final manuscript.

REFERENCES

1. Nair H, Simoes EA, Rudan I*, et al.* Global and regional burden of hospital admissions for severe acute lower respiratory infections in young children in 2010: a systematic analysis. *Lancet*. 2013;**381**:1380-1390.

2. Shi T, McAllister DA, O'Brien KL*, et al.* Global, regional, and national disease burden estimates of acute lower respiratory infections due to respiratory syncytial virus in young children in 2015: a systematic review and modelling study. *Lancet*. 2017;**390**:946-958.

3. Gill PJ, Goldacre MJ, Mant D*, et al.* Increase in emergency admissions to hospital for children aged under 15 in England, 1999–2010: national database analysis. *Arch Dis Child*. 2013.

4. Harron K, Gilbert R, Cromwell D*, et al.* International comparison of emergency hospital use for infants: data linkage cohort study in Canada and England. *BMJ Quality & Safety*. 2017.

5. Moore H, Burgner D, Carville K*, et al.* Diverging trends for lower respiratory infections in non-Aboriginal and Aboriginal children. *J Paediatr Child Health*. 2007;**43**:451-457.

6. Moore HC, Lehmann D, de Klerk N*, et al.* Reduction in disparity for pneumonia hospitalisations between Australian Indigenous and non-Indigenous children. *Journal of epidemiology and community health*. 2012;**66**:489-494.

7. Anderson AJ, Snelling TL, Moore HC*, et al.* Advances in Vaccines to Prevent Viral Respiratory Illnesses in Children. *Paediatr Drugs*. 2017;**19**:523-531.

8. Cakmak S, Hebbern C, Cakmak JD*, et al.* The modifying effect of socioeconomic status on the relationship between traffic, air pollution and respiratory health in elementary schoolchildren. *J Environ Manage*. 2016;**177**:1-8.

9. Moore HC, de Klerk N, Richmond P*, et al.* A retrospective population-based cohort study identifying target areas for prevention of acute lower respiratory infections in children. *BMC Public Health*. 2010;**10**:757.

10. MacIntyre EA, Gehring U, Mölter A*, et al.* Air pollution and respiratory infections during early childhood: an analysis of 10 European birth cohorts within the ESCAPE Project. *Environ Health Perspect*. 2014;**122**:107.

11. Smith KR, Samet JM, Romieu I*, et al.* Indoor air pollution in developing countries and acute lower respiratory infections in children. *Thorax*. 2000;**55**:518-532.

12. Health AGDo. The Australian Immunisation Handbook 10th edition (updated February 2017): Australian Government; 2017.

13. Public Health England. Quarterly vaccination coverage statistics for children aged up to five years in the UK (COVER programme): July to September 20182018 14 Dec 2018.

For peer review only - http://bmjopen.bmj.com/site/about/guidelines.xhtml

14. ABS. 3101.0 - Australian Demographic Statistics, Jun 2014. Australian Bureau of Statistics; 2015; Available from: http://www.abs.gov.au/AUSSTATS/abs@.nsf/Lookup/3101.0Main+Features1Jun%202014?OpenDocument.

15. HealthInfoNet. What details do we know about the Indigenous population? : Australian Indigenous HealthInfoNet 2015; Available from: http://www.healthinfonet.ecu.edu.au/health-facts/health-faqs/aboriginal-population.

16. Holman CD, Bass AJ, Rosman DL*, et al.* A decade of data linkage in Western Australia: strategic design, applications and benefits of the WA data linkage system. *Aust Health Rev*. 2008;**32**:766-777.

17. Kelman CW, Bass AJ, Holman C. Research use of linked health data—a best practice protocol. *Aust N Z J Public Health*. 2002;**26**:251-255.

18. Christenen D, Davis G, Draper G*, et al.* Evidence for the use of an algorithm in resolving inconsistent and missing Indigenous status in administrative data collections. *Australian Journal of Social Issues*. 2014;**49**:423-443.

19. Office for National Statistics. 2011 Census: Population Estimates for the United Kingdom, March 2011. 2012 [6 February 2018]; Available from: https://www.ons.gov.uk/peoplepopulationandcommunity/populationandmigration/populationestimates/bulletins/2011censuspopulationestimatesfortheunitedkingdom/2012-12-17.

20. Herbert A, Wijlaars L, Zylbersztejn A*, et al.* Data Resource Profile: Hospital Episode Statistics Admitted Patient Care (HES APC). *Int J Epidemiol*. 2017;**46**:1093-1093i.

21. NHS Digital. A Guide to Linked Mortality Data from Hospital Episode Statistics and the Office for National Statistics. 2015 [6 February 2018]; Available from: http://content.digital.nhs.uk/media/11668/HES-ONS-Mortality-Data-Guide/pdf/mortality_guide.pdf.

For peer review only - http://bmjopen.bmj.com/site/about/guidelines.xhtml

22. Administrative Data Liaison Service. Birth Registrations - National Records of Scotland. 2016 [4 November 2016]; Available from: http://www.adls.ac.uk/national-records-of-scotland/birth-registrations/?detail.

23. Administrative Data Liaison Service. SMR02 – Maternity Inpatient and Day Case dataset. 2016 [4 November 2016]; Available from: http://www.adls.ac.uk/nhs-scotland/maternity-inpatient-and-day-case-smr02/?detail.

24. Harron K, Gilbert R, Cromwell D*, et al.* Linking Data for Mothers and Babies in De-Identified Electronic Health Data. *PLoS ONE*. 2016;**11**:e0164667.

25. Roberts RF, Innes KC, Walker SM. Introducing ICD-10-AM in Australian hospitals. *The Medical Journal of Australia*. 1998;**169**:S32-35.

26. Pink B. An Introduction to Socio-Economic Indexes for Areas (SEIFA) - Information paper: Australian Bureau of Statistics, Commonwealth of Australia 2008.

27. Smith T, Noble M, Noble S*, et al.* The English Indices of Deprivation 2015: Technical Report: Department for Communities and Local Government 2015.

28. Brown D, Allik M, Dundas R*, et al.* Carstairs Scores for Scottish Postcode Sectors, Datazones & Output Areas from the 2011 Census: University of Glasgow 2014.

29. Gonzalez-Izquierdo A, Cortina-Borja M, Woodman J*, et al.* Maltreatment or violence-related injury in children and adolescents admitted to the NHS: comparison of trends in England and Scotland between 2005 and 2011. *BMJ Open*. 2014;**4**:e004474.

30. StataCorp. Stata Statistical Software. Release 14. College Station, TX.: StataCorp LP.; 2015.

31. Kivistö JE, Protudjer JLP, Karjalainen J*, et al.* Trends in paediatric asthma hospitalisations – differences between neighbouring countries. *Thorax*. 2017.

32. Department of Health. Emergecny admissions to hospital: managing the demand: National Audit Office 2013.

33. Hawker JI, Olowokure B, Sufi F*, et al.* Social deprivation and hospital admission for respiratory infection:: an ecological study. *Respir Med*. 2003;**97**:1219-1224.

For peer review only - http://bmjopen.bmj.com/site/about/guidelines.xhtml

34. Zubrick SR, Lawrence D, Silburn S*, et al.* The Western Australian Aboriginal Child Health Survey: The Health of Aboriginal Children and Young People 1ed. Perth, Western Australia: Telethon Institute for Child Health Research 2004.

35. Holman RC, Hennessy TW, Haberling DL*, et al.* Increasing trend in the rate of infectious disease hospitalisations among Alaska Native people. *Int J Circumpolar Health*. 2013;**72**:20994.

36. O'Grady K-Ann F, Lee Katherine J, Carlin John B*, et al.* Increased risk of hospitalization for acute lower respiratory tract infection among Australian Indigenous infants 5-23 months of age following pneumococcal vaccination: A cohort study. *Clin Infect Dis*. 2010;**50**:970-978.

37. Jardine A, Menzies RI, McIntyre PB. Reduction in Hospitalizations for Pneumonia Associated With the Introduction of a Pneumococcal Conjugate Vaccination Schedule Without a Booster Dose in Australia. *Pediatr Infect Dis J*. 2010;**29**:000-000.

38. Moore HC, Lim FJ, Fathima P*, et al.* Exploring RSV epidemiology in Australian chidlren: estimates of the population burden and effectiveness of a maternal vaccine. **RSV Vaccines for the World**; 29 Nov - 1 Dec; Malaga, Spain2017.

39. Thomas SL, Wakerman J, Humphreys JS. Ensuring equity of access to primary health care in rural and remote Australia - what core services should be locally available? *Int J Equity Health*. 2015;**14**:111.

40. Hardelid P, Verfuerden M, McMenamin J*, et al.* Risk factors for admission to hospital with laboratory-confirmed influenza in young children: birth cohort study. *Eur Respir J*. 2017;**50**:1700489.

41. World Health Organization. RSV Vaccine Research and Develoment Technology Roadmap2017.

42. Spilsbury K, Rosman D, Alan J*, et al.* Cross border hospital use: analysis using data linkage across four Australian states. *Med J Aust*. 2015;**202**:582-586.

43. Moore HC, de Klerk N, Jacoby P*, et al.* Can linked emergency department data help assess the out-of-hospital burden of acute lower respiratory infections? A population-based cohort study. *BMC Public Health*. 2012;**12**:703.

For peer review only - http://bmjopen.bmj.com/site/about/guidelines.xhtml

44. Lim F, Blyth CC, Fathima P, *et al.* Record linkage study of the pathogen-specific burden of respiratory viruses in children. *Influenza & Other Respiratory Viruses*. 2017;**11**:502-510.

For peer review only

Figure legends

Figure 1: Hospitalisation rates for ARI in Western Australia, England and Scotland by level of socio-economic deprivation for A) infants (<1 year) and B) young children (1-4 years). Those in the <10% level represent the most deprived and those ≥ 90% represent those least deprived.

Figure 2: Hospitalisation rates by year of admission for infants (<1 year) and children (1-4 years) in Western Australia, England and Scotland for ARI, bronchiolitis, pneumonia and unspecified ALRI.

For peer review only

Table 1: Number of admissions and hospitalisation rate for ARI by diagnostic category by principal diagnosis in infants aged <1 year and children aged 1-4 years in Western Australia, England and Scotland

Diagnosis	Western Australia				England				Scotland			
	n	(%)	Rate[a]	(95% CI)	n	(%)	Rate[a]	(95% CI)	n	(%)	Rate[a]	(95% CI)
**<1 year[b]**												
Whooping cough	220	(1.6)	0.7	(0.6, 0.8)	2395	(1.1)	0.5	(0.4, 0.5)	372	(1.4)	0.6	(0.5, 0.6)
Pneumonia	1278	(9.4)	4.1	(3.9, 4.4)	15592	(7.2)	2.9	(2.9, 3.0)	1245	(4.8)	1.9	(1.8, 2.1)
Bronchiolitis	10,652	(78.7)	34.4	(33.8, 35.1)	171805	(78.8)	32.2	(32.1, 32.4)	22021	(84.4)	34.3	(33.9, 34.8)
Influenza	407	(3.0)	1.3	(1.2, 1.4)	1627	(0.7)	0.3	(0.3, 0.3)	426	(1.6)	0.7	(0.6, 0.7)
Unspecified ALRI	809	(6.0)	2.6	(2.4, 2.8)	24563	(11.3)	4.6	(4.5, 4.7)	1797	(6.9)	2.8	(2.7, 2.9)
Bronchitis	169	(1.2)	0.5	(0.5, 0.6)	2003	(0.9)	0.4	(0.4, 0.4)	242	(0.9)	0.4	(0.3, 0.4)
All ARI	13,535	(100.0)	43.7	(43.0, 44.5)	217985	(100.0)	40.1	(40.7, 41.1)	26103	(100.0)	40.7	(40.2, 41.2)
**1-4 years[c]**												
Whooping cough	33	(0.4)	0.04	(0.03, 0.06)	95	(0.1)	0.008	(0.007, 0.01)	23	(0.2)	0.01	(0.01,0.02)
Pneumonia	3031	(41.6)	3.7	(3.6, 3.9)	29741	(33.2)	2.5	(2.5, 2.6)	3411	(26.9)	2.1	(2.0, 2.1)
Bronchiolitis	1893	(26.0)	2.3	(2.2, 2.4)	8283	(9.2)	0.7	(0.7, 0.7)	3141	(24.7)	1.9	(1.8, 2.0)
Influenza	366	(5.0)	0.4	(0.4, 0.5)	1714	(1.9)	0.2	(0.1, 0.2)	392	(3.1)	0.2	(0.2, 0.3)
Unspecified ALRI	1767	(24.3)	2.2	(2.1, 2.3)	48910	(54.6)	4.2	(4.1, 4.2)	5570	(43.9)	3.3	(3.3, 3.4)
Bronchitis	195	(2.7)	0.2	(0.2, 0.3)	859	(1.0)	0.1	(0.1, 0.1)	161	(1.3)	0.1	(0.1, 0.1)
All ALRI	7285	(100.0)	9.0	(8.8, 9.2)	89602	(100.0)	7.6	(7.6, 7.7)	12698	(100.0)	7.6	(7.5, 7.8)

[a]Rate is per 1000/child-years

[b]2001-2012 for Western Australia and Scotland; 2004-2012 for England

For peer review only - http://bmjopen.bmj.com/site/about/guidelines.xhtml

For peer review only

[c]2005-2012 for Western Australia and Scotland; 2008-2012 for England

For peer review only

For peer review only - http://bmjopen.bmj.com/site/about/guidelines.xhtml

Table 2: Risk of hospitalisation for bronchiolitis, pneumonia, unspecified ALRI and overall ARI from log-linear modelling in infants aged <1 year in Western Australia, England and Scotland

Exposure	Western Australia		England		Scotland	
	IRR	(95% CI)	IRR	(95% CI)	IRR	(95% CI)
**Bronchiolitis**						
Year[a]	0.99	(0.98, 1.00)	1.05	(1.04, 1.07)	1.04	(1.03, 1.05)
Male	*Reference*		*Reference*		*Reference*	
Female	0.68	(0.64, 0.72)	0.68	(0.63, 0.74)	0.70	(0.64, 0.77)
Deprivation <10%	3.34	(3.02, 3.71)	1.94	(1.73, 2.19)	1.28	(1.16, 1.42)
Deprivation 10-49%	2.04	(1.75, 2.37)	1.48	(1.07, 2.06)	1.29	(0.89, 1.87)
Deprivation 50-89%	1.36	(1.19, 1.55)	1.18	(0.95, 1.45)	1.09	(0.85, 1.40)
Deprivation ≥90%	*Reference*		*Reference*		*Reference*	
Non-ARI admissions	1.00	(1.00, 1.00)	1.00	1.00, 1.00)	1.00	(1.00, 1.00)
**Pneumonia**						
Year[a]	0.94	(0.93, 0.96)	0.98	(0.97, 0.99)	0.97	(0.94, 0.99)
Male	*Reference*		*Reference*		*Reference*	
Female	0.75	(0.67, 0.84)	0.76	(0.70, 0.82)	0.80	(0.67, 0.97)
Deprivation 0-10%	6.91	(5.59, 8.56)	1.47	(1.30, 1.66)	1.09	(0.88, 1.37)
Deprivation 10-49%	3.26	(2.49, 4.28)	0.90	(0.65, 1.25)	0.74	(0.39, 1.43)
Deprivation 50-89%	1.66	(1.29, 2.13)	0.86	(0.70, 1.07)	0.80	(0.51, 1.25)
Deprivation >90%	*Reference*		*Reference*		*Reference*	
Non-ARI admissions	1.00	(1.00, 1.00)	1.00	(1.00, 1.00)	1.00	(1.00, 1.00)
**Unspecified ALRI**						
Year[a]	0.95	(0.93, 0.97)	1.06	(1.05, 1.07)	1.02	(1.00, 1.04)
Male	*Reference*		*Reference*		*Reference*	
Female	0.62	(0.54, 0.71)	0.65	(0.61, 068)	0.73	(0.62, 0.85)
Deprivation <10%	8.90	(6.69, 11.83)	1.81	(1.66, 1.98)	0.93	(0.78, 1.12)
Deprivation 10-49%	4.18	(2.93, 5.96)	1.34	(1.06, 1.70)	0.84	(0.48, 1.48)
Deprivation 50-89%	1.96	(1.40, 2.73)	1.11	(0.95, 1.30)	0.85	(0.58, 1.26)
Deprivation >90%	*Reference*		*Reference*		*Reference*	
Non-ARI admissions	1.00	(1.00, 1.00)	1.00	(1.00, 1.00)	1.00	(1.00, 1.00)
**Total ARI**						
Year[a]	0.99	(0.98, 1.00)	1.05	(1.04,1.06)	1.03	(1.02, 1.04)
Male	*Reference*		*Reference*		*Reference*	

For peer review only - http://bmjopen.bmj.com/site/about/guidelines.xhtml

Female	0.68	(0.65, 0.72)	0.69	(0.64, 0.73)	0.71	(0.65, 0.77)
Deprivation 0-10%	3.85	(3.50, 4.21)	1.87	(1.70, 2.06)	1.25	(1.14, 1.37)
Deprivation 10-49%	2.22	(1.95, 2.54)	1.41	(1.07, 1.85)	1.25	(0.88, 1.77)
Deprivation 50-89%	1.42	(1.26, 1.60)	1.14	(0.96, 1.36)	1.08	(0.85, 1.36)
Deprivation >90%	*Reference*		*Reference*		*Reference*	
Non-ARI admissions	1.00	(1.00, 1.00)	1.00	(1.00, 1.00)	1.00	(1.00, 1.00)

3 IRR, Incidence Rate Ratio

4 [a]Year included as a linear term

5

For peer review only

For peer review only - http://bmjopen.bmj.com/site/about/guidelines.xhtml

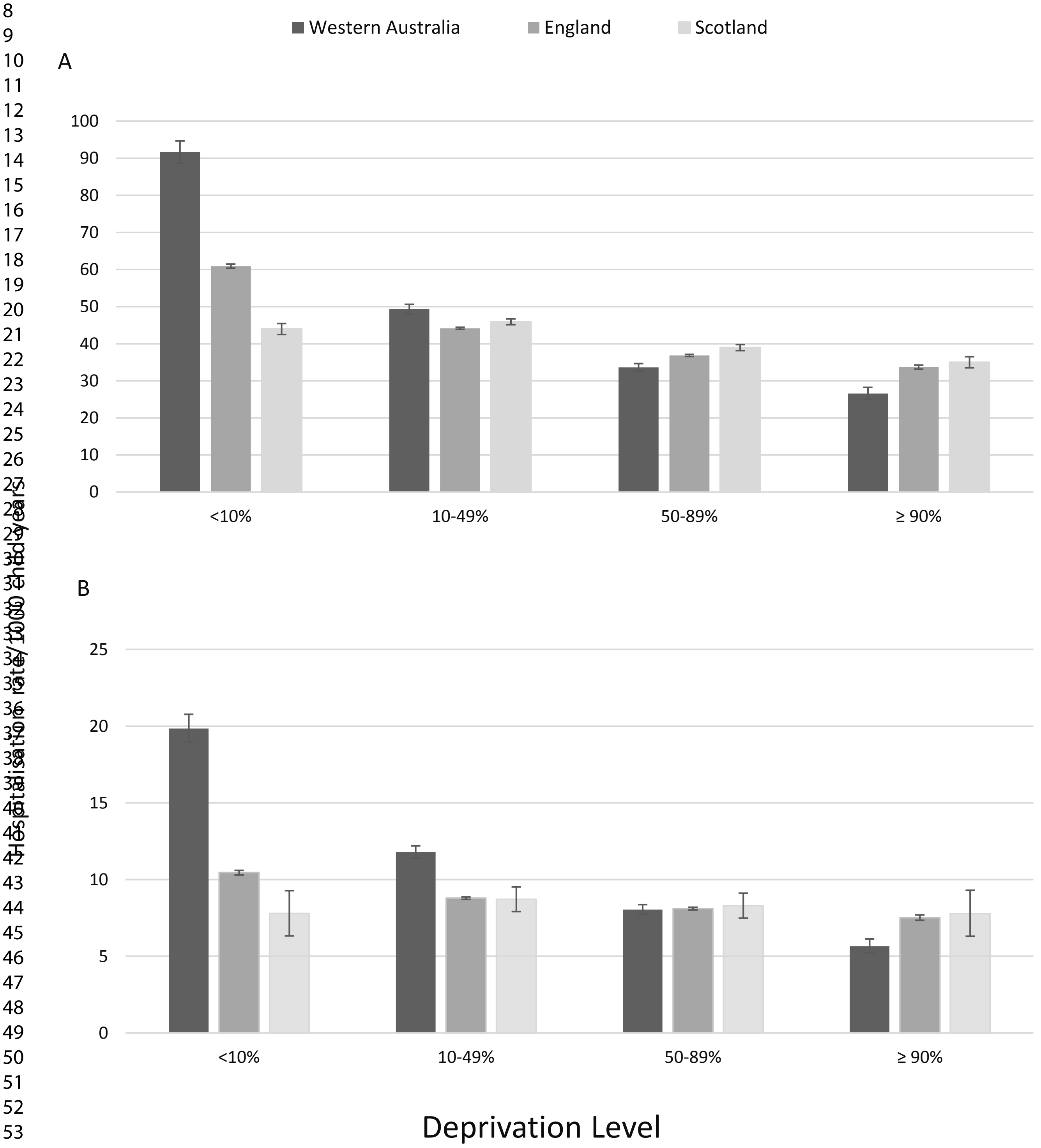
Western Australia
England
Scotland
A
100
90
80
70
60
50
40
30
20
10
0
<10%
10-49%
50-89%
≥ 90%
Hospitalisation rate/1000 child years
B
25
20
15
10
5
0
<10%
10-49%
50-89%
≥ 90%
Deprivation Level

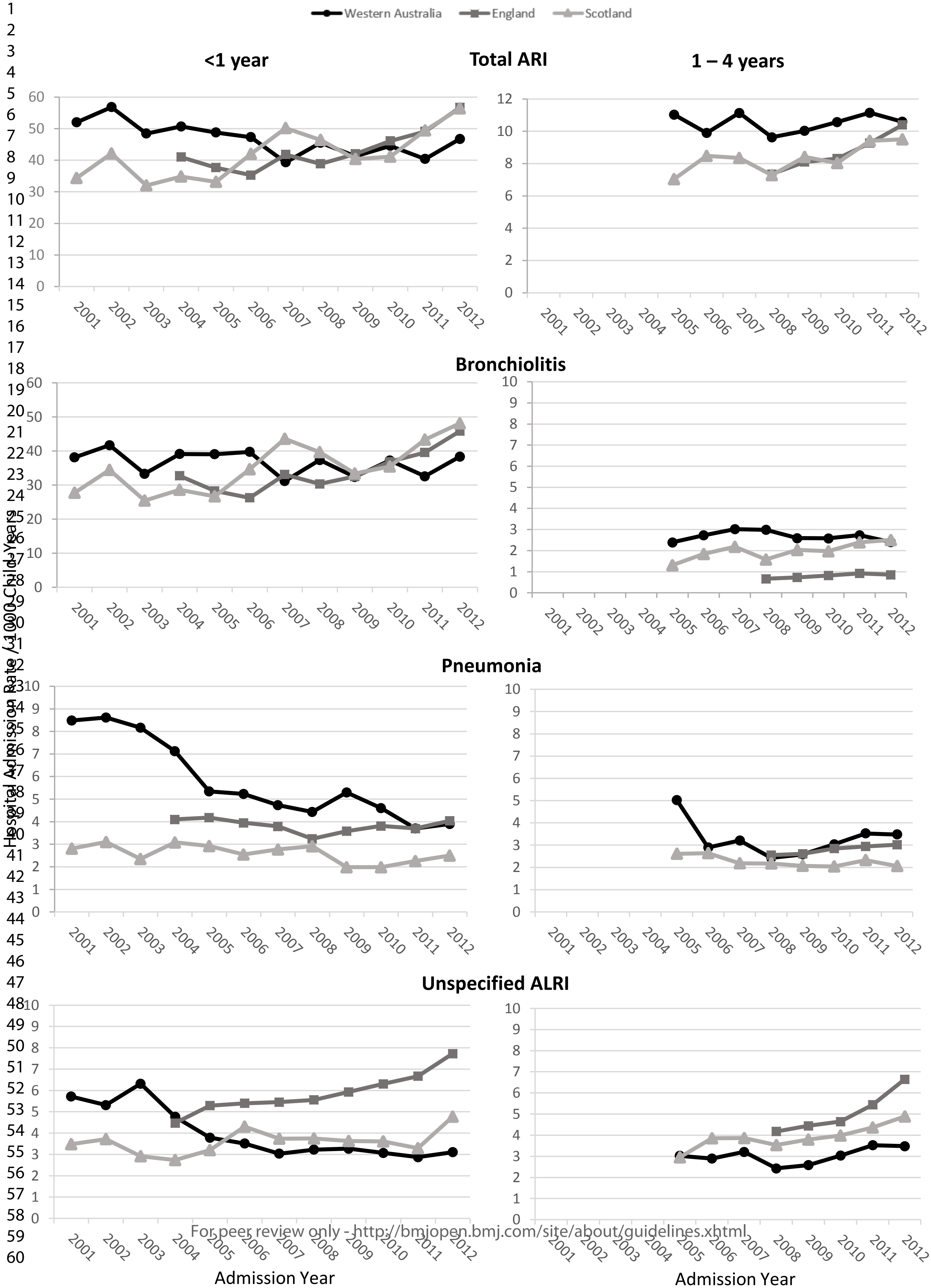
Western Australia
England
Scotland
<1 year
Total ARI
1 – 4 years
Bronchiolitis
Pneumonia
Unspecified ALRI
Hospital Admission Rate / 1000 Child-Years
Admission Year
Admission Year

Supplementary Table 1: Cohort characteristics by jurisdiction

Characteristic		Western Australia		England		Scotland	
		n	(%)	n	(%)	n	(%)
Sex[a]	Male	173,081	(51.2)	3,044,931	(51.3)	359,159	(51.3)
	Female	164,828	(48.8)	2,894,078	(48.7)	340,430	(48.7)
Year of birth	2000	22,551	(6.7)	-		51,479	(7.4)
	2001	22,461	(6.7)	-		50,295	(7.2)
	2002	22,412	(6.7)	-		49,425	(7.1)
	2003	22,345	(6.6)	535,724	(9.0)	50,450	(7.2)
	2004	23,361	(6.9)	551,939	(9.3)	52,417	(7.5)
	2005	24,776	(7.3)	560,894	(9.4)	52,415	(7.5)
	2006	26,627	(7.9)	579,220	(9.8)	53,830	(7.7)
	2007	26,943	(8.0)	586,526	(9.9)	55,765	(8.0)
	2008	28,216	(8.4)	614,471	(10.4)	57,481	(8.2)
	2009	28,588	(8.5)	611,427	(10.3)	57,007	(8.2)
	2010	28,565	(8.5)	629,778	(10.7)	56,586	(8.1)
	2011	29,535	(8.7)	632,306	(10.7)	56,510	(8.1)
	2012	31,529	(9.3)	636,724	(10.7)	55,929	(8.0)
Deprivation[b]	<10% (most deprived)	40,936	(12.1)	885,891	(14.9)	85,987	(12.3)
	10-49%	122,309	(36.2)	2,635,562	(44.4)	295,372	(42.2)
	50-89%	131,232	(38.8)	1,957,789	(33.0)	252,407	(36.1)
	≥90% (least deprived)	43,432	(12.9)	459,767	(7.6)	65,823	(9.4)
**TOTAL**		**337,909**	**(100.0)**	**5,939,009**	**(100.0)**	**699,589**	**(100.0)**

[a] Sex was missing was 7205 children (England) with no missing data from Western Australia or Scotland.

[b] Deprivation scores were missing for 18,920 children (Western Australia), 146,588 (England) and 827 (Scotland).

For peer review only

Supplementary Table 2: Hospitalisation rate for ARI by diagnostic category by primary diagnosis only (PDx) and primary diagnosis plus all additional diagnoses (Any Dx) in infants aged <1 year and children aged 1-4 years in Western Australia, England and Scotland

	Western Australia			England			Scotland		
	Rate[a]		IRR (95% CI)	Rate[a]		IRR (95% CI)	Rate[a]		IRR (95% CI)
**ARI diagnosis**	**Any Dx**	**PDx**		**Any Dx**	**PDx**		**Any Dx**	**PDx**	
**<1 year[b]**									
Whooping cough	0.8	0.7	1.2 (1.0, 1.4)	0.5	0.5	1.1 (1.0, 1.2)	0.6	0.6	1.1 (0.9, 1.2)
Pneumonia	5.6	4.1	1.4 (1.3, 1.5)	3.8	2.9	1.3 (1.3, 1.3)	2.6	1.9	1.3 (1.2, 1.4)
Bronchiolitis	36.6	34.4	1.1 (1.0, 1.1)	34.2	32.2	1.1 (1.1, 1.1)	35.4	34.3	1.0 (1.0, 1.1)
Influenza	1.6	1.3	1.3 (1.1, 1.4)	0.4	0.3	1.4 (1.3, 1.5)	0.9	0.7	1.3 (1.2, 1.5)
Unspecified ARI	3.9	2.6	1.5 (1.4, 1.6)	5.9	4.6	1.3 (1.3, 1.3)	3.6	2.8	1.3 (1.2, 1.4)
Bronchitis	0.7	0.5	1.3 (1.1, 1.6)	0.5	0.4	1.2 (1.1, 1.3)	0.5	0.4	1.2 (1.1, 1.5)
Total ARI	46.4	43.7	1.1 (1.0, 1.1)	43.4	40.1	1.1 (1.1, 1.1)	42.1	40.7	1.0 (1.0, 1.1)
**1-4 years[c]**									
Whooping cough	0.05	0.04	1.1 (0.7, 1.9)	0.009	0.008	1.1 (0.9, 1.5)	0.02	0.01	1.1 (0.7, 2.0)
Pneumonia	4.3	3.7	1.2 (1.1, 1.2)	2.8	2.5	1.1 (1.1, 1.1)	2.3	2.1	1.1 (1.1, 1.1)
Bronchiolitis	2.7	2.3	1.2 (1.1, 1.2)	0.8	0.7	1.1 (1.1, 1.2)	2.0	1.9	1.1 (1.0, 1.1)
Influenza	0.6	0.4	1.3 (1.1, 1.4)	0.2	0.2	1.3 (1.2, 1.4)	0.3	0.2	1.2 (1.1, 1.4)
Unspecified ARI	3.0	2.2	1.4 (1.3, 1.5)	5.1	4.2	1.2 (1.2, 1.2)	3.9	3.3	1.2 (1.1, 1.2)
Bronchitis	0.3	0.2	1.4 (1.2, 1.7)	0.1	0.1	1.2 (1.1, 1.3)	0.1	0.1	1.3 (0.0, 1.6)
Total ARI	10.5	9.0	1.2 (1.1, 1.2)	8.7	7.6	1.1 (1.1, 1.1)	8.4	7.6	1.1 (0.0, 1.1)

IRR, incidence rate ratio

For peer review only

[a]Rate is per 1000/child-years

[b]2001-2012 for Western Australia and Scotland; 2004-2012 for England

[c]2005-2012 for Western Australia and Scotland; 2008-2012 for England

For peer review only

STROBE Statement—Checklist of items that should be included in reports of ***cohort studies***

	Item No	Recommendation	Page No
**Title and abstract**	1	(*a*) Indicate the study's design with a commonly used term in the title or the abstract	1
		(*b*) Provide in the abstract an informative and balanced summary of what was done and what was found	2
**Introduction**			
Background/rationale	2	Explain the scientific background and rationale for the investigation being reported	4
Objectives	3	State specific objectives, including any prespecified hypotheses	5
**Methods**			
Study design	4	Present key elements of study design early in the paper	5
Setting	5	Describe the setting, locations, and relevant dates, including periods of recruitment, exposure, follow-up, and data collection	5-6
Participants	6	(*a*) Give the eligibility criteria, and the sources and methods of selection of participants. Describe methods of follow-up	5-6
		(*b*) For matched studies, give matching criteria and number of exposed and unexposed	
Variables	7	Clearly define all outcomes, exposures, predictors, potential confounders, and effect modifiers. Give diagnostic criteria, if applicable	6-7
Data sources/ measurement	8*	For each variable of interest, give sources of data and details of methods of assessment (measurement). Describe comparability of assessment methods if there is more than one group	7
Bias	9	Describe any efforts to address potential sources of bias	8
Study size	10	Explain how the study size was arrived at	6
Quantitative variables	11	Explain how quantitative variables were handled in the analyses. If applicable, describe which groupings were chosen and why	7-8
Statistical methods	12	(*a*) Describe all statistical methods, including those used to control for confounding	8
		(*b*) Describe any methods used to examine subgroups and interactions	8
		(*c*) Explain how missing data were addressed	8, Additional File 1
		(*d*) If applicable, explain how loss to follow-up was addressed	
		(*e*) Describe any sensitivity analyses	
**Results**			
Participants	13*	(a) Report numbers of individuals at each stage of study—eg numbers potentially eligible, examined for eligibility, confirmed eligible, included in the study, completing follow-up, and analysed	9
		(b) Give reasons for non-participation at each stage	
		(c) Consider use of a flow diagram	
Descriptive data	14*	(a) Give characteristics of study participants (eg demographic, clinical, social) and information on exposures and potential confounders	Additional File 1
		(b) Indicate number of participants with missing data for each variable of interest	Additional File 1
		(c) Summarise follow-up time (eg, average and total amount)	9
Outcome data	15*	Report numbers of outcome events or summary measures over time	9

For peer review only - http://bmjopen.bmj.com/site/about/guidelines.xhtml

Main results	16	(*a*) Give unadjusted estimates and, if applicable, confounder-adjusted estimates and their precision (eg, 95% confidence interval). Make clear which confounders were adjusted for and why they were included<br>(*b*) Report category boundaries when continuous variables were categorized<br>(*c*) If relevant, consider translating estimates of relative risk into absolute risk for a meaningful time period	23-26
Other analyses	17	Report other analyses done—eg analyses of subgroups and interactions, and sensitivity analyses	13
**Discussion**			
Key results	18	Summarise key results with reference to study objectives	11
Limitations	19	Discuss limitations of the study, taking into account sources of potential bias or imprecision. Discuss both direction and magnitude of any potential bias	14
Interpretation	20	Give a cautious overall interpretation of results considering objectives, limitations, multiplicity of analyses, results from similar studies, and other relevant evidence	13-14
Generalisability	21	Discuss the generalisability (external validity) of the study results	14-15
**Other information**			
Funding	22	Give the source of funding and the role of the funders for the present study and, if applicable, for the original study on which the present article is based	16

*Give information separately for exposed and unexposed groups.

Note: An Explanation and Elaboration article discusses each checklist item and gives methodological background and published examples of transparent reporting. The STROBE checklist is best used in conjunction with this article (freely available on the Web sites of PLoS Medicine at http://www.plosmedicine.org/, Annals of Internal Medicine at http://www.annals.org/, and Epidemiology at http://www.epidem.com/). Information on the STROBE Initiative is available at http://www.strobe-statement.org.

For peer review only

BMJ Open

Temporal trends and socio-economic differences in acute respiratory infection hospitalisations in children: an inter-country comparison of birth cohort studies in Western Australia, England and Scotland

Journal:	*BMJ Open*
Manuscript ID	bmjopen-2018-028710.R2
Article Type:	Research
Date Submitted by the Author:	03-Apr-2019
Complete List of Authors:	Moore, Hannah; Wesfarmers Centre of Vaccines and Infectious Diseases, Telethon Kids Institute, The University of Western Australia,<br>de Klerk, Nicholas; Telethon Kids Institute, Wesfarmers Centre of Vaccines and Infectious Diseases<br>Blyth, Christopher C.; Division of Paediatrics, School of Medicine, The University of Western Australia; PathWest Laboratory Medicine WA, Perth Children's Hospital<br>Gilbert, Ruth; University College London, Institute of Child Health<br>Fathima, Parveen; Wesfarmers Centre of Vaccines and Infectious Diseases, Telethon Kids Institute, The University of Western Australia<br>Zylbersztejn, Ania; University College London Great Ormond Street Institute of Child Health , Population, policy and practice<br>Verfürden, Maximiliane ; University College London, Great Ormond Street Institute of Child Health<br>Hardelid, Pia; UCL Institute of Child Health, Centre for Paediatric Epidemiology and Biostatistics
**Primary Subject Heading**:	Epidemiology
Secondary Subject Heading:	Paediatrics, Infectious diseases
Keywords:	Respiratory infections < THORACIC MEDICINE, socio-economic disparity, infant, hospitalisation, record linkage

SCHOLARONE™ Manuscripts

Temporal trends and socio-economic differences in acute respiratory infection hospitalisations in children: an inter-country comparison of birth cohort studies in Western Australia, England and Scotland

Hannah C Moore[1], Nicholas de Klerk[1], Christopher C Blyth[1, 2, 3, 4], Ruth Gilbert[5], Parveen Fathima[1], Ania Zylbersztejn[5], Maximiliane Verfürden[5], Pia Hardelid[5]

[1]Wesfarmers Centre of Vaccines & Infectious Diseases, Telethon Kids Institute, The University of Western Australia, Perth, Australia

[2]School of Medicine, The University of Western Australia, Perth, Australia

[3]Department of Infectious Diseases, Princess Margaret Hospital for Children, Perth, Australia

[4]PathWest Laboratory Medicine WA, QE11 Medical Centre, Perth, Australia

[5]University College London Great Ormond Street Institute of Child Health, London, United Kingdom

Author for Correspondence:

Hannah C Moore, Wesfarmers Centre of Vaccines & Infectious Diseases, Telethon Kids Institute, The University of Western Australia. PO Box 855, West Perth, Western Australia 6872

Email: hannah.moore@telethonkids.org.au

Phone: + 61 8 6319 1427 / +61 409 100 007

For peer review only - http://bmjopen.bmj.com/site/about/guidelines.xhtml

ABSTRACT

Objectives: Acute respiratory infections (ARI) are a global cause of childhood morbidity. We compared temporal trends and socioeconomic disparities for ARI hospitalisations in young children across Western Australia, England and Scotland.

Design: Retrospective population-based cohort studies using linked birth, death and hospitalisation data.

Setting and Participants: Population birth cohorts spanning 2000-2012 (Western Australia and Scotland) and 2003-2012 (England).

Outcome Measures: ARI hospitalisations in infants (<12 months) and children (1-4 years) were identified through ICD-10 diagnosis codes. We calculated admission rates per 1000 child-years by diagnosis and jurisdiction- specific socio-economic deprivation and used negative binomial regression to assess temporal trends.

Results: The overall infant ARI admission rate was 44.3/1000 child-years in Western Australia, 40.7/1000 in Scotland and 40.1/1000 in England. Equivalent rates in children aged 1-4 years were 9.0, 7.6, and 7.6. Bronchiolitis was the most common diagnosis. Compared with the least socio-economically deprived, those most deprived had higher ARI hospitalisation risk (incidence rate ratio 3.9 [95% confidence interval 3.5, 4.2] for Western Australia; 1.9 [1.7, 2.1] for England; 1.3 [1.1, 1.4] for Scotland). ARI admissions in infants were stable in Western Australia but increased annually in England (5%) and Scotland (3%) after adjusting for non-ARI admissions, sex, and deprivation.

Conclusions: Admissions for ARI were higher in Western Australia and displayed greater socio-economic disparities than England and Scotland, where ARI rates are increasing. Prevention programs focusing on disadvantaged populations in all three countries are likely to translate into real improvements in the burden of ARI in children.

For peer review only

For peer review only - http://bmjopen.bmj.com/site/about/guidelines.xhtml

Keywords (3-10)

Acute respiratory infections; hospitalisation; socio-economic disparity; international comparison; infant; population; record linkage

ARTICLE SUMMARY

Strengths and limitations of this study

- We used population-level data from three countries to assess hospitalisation rates and changes over time for acute respiratory infections in children
- Analysis protocols and diagnosis coding was standardised across each country
- Hospitalisation rates for acute respiratory infections were described according to level of socio-economic deprivation
- To control for changing admission thresholds within each country, we adjusted our models for all non-acute respiratory infection admissions
- A limitation of this study is the different measures of socio-economic deprivation available across the three countries

For peer review only

BACKGROUND

Acute respiratory infections (ARI) including bronchiolitis, pneumonia and influenza are a major cause of hospitalisation in children worldwide, responsible for approximately 12 million annual episodes in children under 5 years of age[1, 2]. In England, the hospital admission rate for ARI increased by 40% from 1999-2010 among children aged less than 15 years[3] and bronchiolitis was the most common reason for unplanned admissions in infants from 2010-2013.[4] While hospitalisations for ARI doubled from 1992-2000 in Western Australia,[5] they since stabilised 2000-2005.[6] Vaccination programmes including influenza, pertussis and pneumococcal disease have been implemented in North America, Europe and Australia, but the majority of ARI hospitalisations in high income countries are now caused by non-vaccine preventable viruses including Respiratory Syncytial Virus (RSV), Parainfluenza virus and Human Metapneumovirus.[7]

ARI hospitalisations are more common among children from poorer socio-economic backgrounds.[8, 9] In addition to access to inadequate health care, risk factors for developing severe symptoms of ARIs, including prematurity, low birth weight, congenital anomalies, exposure to environmental tobacco smoke, damp and mould, and household overcrowding are all more common among children growing up in more deprived families in both high and low income settings.[10, 11] Understanding the impact of socio-economic disparities on ARI hospitalisations among children (both over time and between countries) can provide an estimate of the preventable proportion of ARI. Linkage of administrative health datasets provides a platform to investigate these trends in populations over many years. Additionally, the availability of comparable hospital admission datasets with similar coding systems using International Classification of Diseases, 10th edition (ICD-10) diagnosis codes allows comparison of hospitalisation rates among children for ARI according to deprivation level.

For peer review only - http://bmjopen.bmj.com/site/about/guidelines.xhtml

Using record linkage resources within Western Australia, England and Scotland, we conducted a comparative analysis of the three jurisdictions to investigate the hospitalisation rates for ARI in children aged less than 5 years. All three jurisdictions have publicly funded health care with free access to primary and public hospital care. Each jurisdiction has established childhood vaccination programs targeting acute respiratory infections. This includes diphtheria, tetanus, pertussis, *Haemophilus influenzae* type B (3 dose infant schedule), pneumococcal disease (2 + 1 schedule) and recently, seasonal influenza. Excluding influenza, vaccination coverage at age 12 months is >90% for all 3 jurisdictions.[12, 13] Our aim was to compare population-based hospitalisation rates by ARI diagnosis, age and level of socio-economic deprivation, and assess how ARI hospitalisation rates have changed over time.

METHODS

Data Sources and Study Populations

We conducted separate population-based birth cohort studies using administrative data from Western Australia, England and Scotland. Western Australia covers the western third of Australia, an area of 2.5 million square kilometres with a population of nearly 2.6 million,[14] 3.6% of whom identify as being Aboriginal and/or Torres Strait Islander (herein referred to as Aboriginal).[15] Births were identified from the Midwives' Notification System and Birth Register, deaths were identified from the Death Register and hospitalisations were recorded in the Hospital Morbidity Database Collection that provides full coverage of all hospital separations (hereafter referred to as hospitalisations). In the absence of a unique person identifier in Australia, extracted data were probabilistically linked by the Western Australian Data Linkage Branch using a series of demographic identifiers using an established best practice protocol.[16, 17] Aboriginal status was derived using a

For peer review only - http://bmjopen.bmj.com/site/about/guidelines.xhtml

validated algorithm using Aboriginal identification information across all available records.[18] England has a population of 53.9 million.[19] The birth cohort was established by linking hospital birth and delivery records from the Hospital Episode Statistics (HES) database.[20] Hospitalisations and deaths were identified via linkage to mortality registration data from the Office for National Statistics.[21] Data linkage in England was carried out by NHS Digital, using a deterministic algorithm based on the NHS number (a unique patient identifier in the English NHS), postcode, date of birth, sex and local hospital numbers. Scotland has a population of 5.3 million.[19] The Scottish birth cohort was developed through linking data from birth registration and maternity databases[22, 23]. Hospitalisations and deaths were identified via linkage to the Scottish Morbidity Record 01 (SMR-01) and mortality records using deterministic linkage carried out by the electronic Data Research and Innovation Service (eDRIS) based on the Community Health Index number, a unique identifier recorded on all births and subsequent encounters within the Scottish NHS.

The datasets represented 99.9% of all births in Western Australia, 97.5% in England[24] and 100% in Scotland with full coverage of inpatient and day admissions. Our study population comprised of singleton births in Western Australia and Scotland 2000-2012 and England 2003-2012. Multiple births were excluded due to a higher likelihood of linkage error. Children were followed from birth until their fifth birthday, date of death, or 30 June 2013 (the end of follow-up) or (Scotland only) date of emigration, whichever occurred first.

Outcome Measures

Our outcome measure was an ARI emergency hospitalisation for children in their first 5 years of life. All inter-hospital transfers were collapsed into a single admission. We identified hospitalisations for ARI using a selection of ICD-10 diagnosis codes (ICD-10-AM for Western Australia).[25]

For peer review only - http://bmjopen.bmj.com/site/about/guidelines.xhtml

Hospitalisation data for each jurisdiction provided a principal diagnosis code and up to 20 secondary diagnosis codes in Western Australia, 19 in England and 5 in Scotland. We identified ARI hospitalisations using the principal diagnosis code and all the available additional diagnosis codes as six diagnostic groups: whooping cough (A37), influenza (J09-J11), pneumonia (J12-J18, B59, B05.2, B37.1, B01.2), bronchitis (J20, J40), bronchiolitis (J21) and unspecified acute lower respiratory infection.(J22) Consistent with our previous Western Australian work,[6] ARI hospitalisations within 14 days of a previous ARI hospitalisation were classified as a single infection episode. In such cases we applied a hierarchical diagnosis algorithm[6] within the readmission set in order to code an overall principal diagnosis. This algorithm ranked diagnoses in order of disease severity: whooping cough, pneumonia, bronchiolitis, influenza, unspecified ALRI and bronchitis. Children with missing data on sex or deprivation were excluded from the analyses. Deaths due to ARI in these populations are rare and our data would be not sufficiently powered to assess mortality rates in this cohort, especially for Western Australia and Scotland. As such we do not report ARI-related mortality rates here and focus our outcome measure on ARI-related hospitalisations.

For peer review only

Exposure Measures

It is known that hospitalisation rates for ARI are higher in infants aged less than 12 months than those aged older than 12 months. Thus, we assessed hospitalisations for ARI in infants aged less than 12 months and young children aged 1- 4 years at time of admission.Other exposure measures of interest were sex, level of socio-economic deprivation and admission year. In Western Australia, socio-economic deprivation was measured through the Index of Relative Disadvantage (IRSAD), one of the four Socio-Economic Indexes for Areas (SEIFA) derived by the Australian Bureau of Statistics.[26] The IRSAD score is derived from 17 different variables including low income, internet connection, unemployment and education.[26] Scores were grouped into Collectors District, the smallest unit for population-based analyses which, on average, consist of approximately 200 dwellings. For England,

For peer review only - http://bmjopen.bmj.com/site/about/guidelines.xhtml

socio-economic deprivation was measured through the Index of Multiple Deprivation (IMD), based on seven domains of deprivation including income, employment, education, crime, barriers to housing and living environment.[27] IMD scores are measured at Lower Super Output Area Level, covering an average of 1200-1500 households. For Scotland, deprivations scores were based on the Carstairs Index, based on four variables including car ownership, male unemployment, overcrowding and low occupational social class. The Carstairs Index is measured at postcode sector level, which contains an average of 5000 people[28] In all jurisdictions socio-economic deprivation scores were based on mother's residential address at time of her child's delivery and were grouped into four levels based on a country level ranking with the lowest scores representing the most socio-economically deprived.

Statistical Analysis

Consistent methodology was applied to the assembled datasets in the three jurisdictions. We calculated hospitalisation rates per 1000 child-years at risk for each diagnostic grouping of ARI (as principal diagnosis). To assess the impact of including additional diagnosis codes, we compared hospitalisation rates derived using the principal diagnosis code only with rates derived from using the principal plus all additional diagnosis codes (any diagnosis). We used any diagnosis to assess ARI rates by socio-economic deprivation and year of admission. We present age-specific hospitalisation rates with 95% confidence intervals (CI) and where appropriate, rates were compared using incidence rate ratios (IRRs) with 95% CIs. To assess temporal trends, we plotted annual hospitalisation rates in the two age groups for each jurisdiction by admission year for all ARIs and bronchiolitis, pneumonia and unspecified ALRI's. We also used negative binomial regression models to assess linear temporal trends in infant hospitalisations from 2001-2012 (Western Australia and Scotland) and 2004-2012 (England). Year of admission was included as a linear term in the models, and the natural logarithm of child-years at risk was included as an offset in the models. Trends over

For peer review only - http://bmjopen.bmj.com/site/about/guidelines.xhtml

time in ARI admission rates were assumed to be statistically significant if the Wald test *p*-value for the coefficient for the linear year term was <0.05. Models were adjusted for sex and the 4-level socioeconomic indicator and we present IRR's with 95% CI's. In order to control for overall trends in hospitalisation we also adjusted the models for the number of all non-ARI emergency admissions.[29] All data analyses were conducted within each jurisdiction in Stata version 14.0.[30]

Public and Patient Involvement

A community reference group located in Western Australia was consulted during the conduct of this study. No individual patients were involved.

RESULTS

A total of 337,909 (Western Australia), 5,939,009 (England) and 699,590 (Scotland) births were included in the study (Supplementary Table 1). There were 14,480 infant hospitalisations for ARI as a principal diagnosis in Western Australia, 217,985 for England and 26,103 for Scotland giving overall infant hospitalisation rates of 44.3/1000 child-years for Western Australia, 40.7/1000 for Scotland and 40.1/1000 for England. In all jurisdictions, bronchiolitis had the highest hospitalisation rates accounting for 79% of ARI admissions in infants in Western Australia, 79% in England, and 84% in Scotland (Table 1). ARI hospitalisation rates in infants were higher in Western Australia compared with England and Scotland across all ARI diagnoses, most notably for pneumonia, where rates were 1.4-2.2 times higher compared to England and Scotland. The only exception was for unspecified ALRI where the hospitalisation rate in infants were 70% higher in England than in Scotland and Western Australia. ARI hospitalisation rates in children aged 1-4 years were 19% higher in Western Australia compared with England and Scotland (Table 1). The most common ARI principal diagnosis among children aged 1-4 years was pneumonia in Western Australia (42%) and unspecified ALRI in England

For peer review only - http://bmjopen.bmj.com/site/about/guidelines.xhtml

(54.6%) and Scotland (43.9%). Consequently, hospitalisation rates for pneumonia in Western Australian children aged 1-4 years were 1.5-1.8 times higher than England and Scotland.

When ARI hospitalisations were identified based on any diagnosis compared with principal diagnosis only, the difference in hospitalisation rates varied across diagnoses with the most notable difference for unspecified ALRI in Western Australia where rates were 1.5 (95% CI: 1.4, 1.6) times higher in infants when using any diagnosis compared with principal diagnosis only (Supplementary Table 2).

ARI hospitalisation rates were higher for children from the most socio-economically deprived areas. The association with deprivation was greatest in Western Australia and more marked in infants compared to young children aged 1-4 years (Figure 1). The relative difference in ARI hospitalisation rates between the most and least deprived infants was 3.5 (95% CI: 3.2, 3.7) in Western Australia; 1.8 for England and 1.3 for Scotland with similar patterns in children aged 1-4 years (Figure 1). In multivariable models, level of socio-economic deprivation was significantly associated with all ARI categories in all infants but most notably in Western Australia, and in particular, pneumonia (IRR 6.9, 95% CI: 5.6, 8.6) and unspecified ALRI (IRR 8.9, 95% CI: 6.7, 11.8; Table 2).

Overall, ARI hospitalisation rates have increased in England and Scotland, but declined (infants) or remained stable (children aged 1-4 years) in Western Australia (Figure 2). After adjusting for sex, deprivation and non-ARI emergency hospitalisations, the ARI hospitalisation rate among infants increased by 5% per year in England (IRR 1.05, 95% CI: 1.04, 1.07) and by 3% per year (IRR 1.03, 95% CI: 1.02, 1.04) in Scotland with no statistically significant trend in Western Australia (IRR 0.99, 95% CI: 0.98, 1.00; Table 2, Figure 2). Similar results were seen for bronchiolitis admissions in infants.

For peer review only - http://bmjopen.bmj.com/site/about/guidelines.xhtml

For peer review only

Diverging trends were seen with pneumonia and unspecified ALRI across the three jurisdictions with pneumonia hospitalisation rates in infants declining in Western Australia from 9.0/1000 in 2002 to 3.9/1000 in 2012 while rates remained steady around 3-4/1000 in England and 2-3/1000 in Scotland (Figure 2). After adjusting for sex, socio-economic deprivation and non-ARI admissions, the annual decline in pneumonia hospitalisations was 6% in Western Australia (IRR 0.94, 95%CI: 0.93, 0.96), 2% in England and 3% in Scotland (Table 2). Unspecified ALRI declined in Western Australia annually by 5% but increased by 6% and 2% annually in England and Scotland (Table 2).

DISCUSSION

ARI, particularly bronchiolitis, continues to be an important cause of infant and childhood hospitalisation. The availability of linked administrative data in three economically similar jurisdictions with publicly funded healthcare systems afforded us the opportunity to compare ARI hospitalisation rates in children. Overall, admission rates were highest in Western Australia and decreasing or remaining stable but increasing in England and Scotland. The relative differences in ARI admission rates between children from the most socioeconomically deprived areas to the least deprived areas were largest in Western Australia.

The interpretation of hospitalisation trends across countries is complex. We have found higher rates of ARI admissions in Western Australia compared with England and Scotland which could mean a higher incidence in ARI, a higher risk of developing more severe symptoms, or differences in diagnostic coding or hospital admission thresholds. A recent study comparing admission rates between England and Ontario finding substantially higher rates in England was partly explained by differing admission thresholds from differential waiting practices and policies in emergency

For peer review only - http://bmjopen.bmj.com/site/about/guidelines.xhtml

departments.[4] Comparisons of asthma admissions from national hospital data in Finland and Sweden noted diverging trends citing differences in national coding guidelines and subsequent altered admission thresholds.[31] In an attempt to control for changing admissions thresholds over time within each jurisdiction, we adjusted our multivariable models for the overall trend in non-ARI emergency hospital use. However we could not adjust for differing thresholds between countries. Emergency hospitalisations are increasing at a faster rate in England compared to other parts of the United Kingdom[32] and our data here suggests that hospitalisations due to unspecified ALRI and bronchiolitis in England are contributing to that increase. It is also possible that diverging trends are a result of diagnostic shifts in that for the same clinical presentations, a diagnosis of unspecified ALRI is given in England while other non-specific codes (including codes we have not assessed) are given in Western Australia and Scotland. The use of additional diagnosis codes for ARI seemed more frequent in Western Australia compared with England and Scotland and should be taken into consideration for future comparative studies using ICD diagnosis codes.

Hospitalisation rates for ARI were significantly associated with level of socio-economic deprivation, consistent with an earlier analysis in England.[33] This association was strongest in Western Australia with IRRs for those in the most deprived level in the order of 3.9 for all ARIs, up to 8.9 for unspecified ALRI. There appeared a linear relationship with level of deprivation and rates of ARI in Western Australia while rates in all levels (bar the most deprived) not differing in England and Scotland. Western Australian data were inclusive of Aboriginal children, an Indigenous population with higher levels of socio-economic disadvantage[34] compared to their non-Aboriginal peers and a significantly higher burden of pneumonia worldwide,[6, 35, 36] despite reductions in the 2000's and further reductions seen in our results here, most likely due to the positive impact of pneumococcal vaccination.[6, 37] This most likely explains the higher rates of pneumonia seen in Western Australia compared with England and Scotland. We have previously reported that hospitalisation rates for all

For peer review only - http://bmjopen.bmj.com/site/about/guidelines.xhtml

acute respiratory infections are 5 to 7 times higher in young Aboriginal children compared with non-Aboriginal children.[9] Aboriginal children also suffer a disproportionate burden of RSV,[38] the major cause of bronchiolitis which could explain the higher bronchiolitis rates in Western Australia than in England and Scotland. However level of socio-economic deprivation has been associated with hospitalisations for respiratory infections in both Aboriginal and non-Aboriginal children[9] so the contribution of Aboriginal children alone cannot explain the higher socio-economic disparities seen here. Indeed, when Aboriginal children were removed from the analysis, the socio-economic disparities remained, although slightly lessened, and were still higher than England and Scotland (e.g. the IRR for most deprived children for all ARI reduced from 3.9 to 2.1 and for unspecified ALRI reduced from 8.9 to 2.9 (data not shown)). Respiratory infections continue to be a source of health inequalities among disadvantaged children worldwide. Geographical remoteness is more of an issue in Western Australia due to its sheer geographical size in comparison to England and Scotland. The lack of adequate primary care in rural and remote Australia[39] which is often coupled with lower socio-economic levels could be driving higher hospitalisation rates. Nevertheless, these important findings highlight the need for targeted prevention programs such as smoking cessation, improved housing and timely vaccination for key respiratory pathogens for the most disadvantaged populations in all three jurisdictions.

Since 2013, the United Kingdom had been rolling out a universal seasonal influenza vaccination program for children aged 2 years to 16 years and from 2018, all Australian states and territories offer free seasonal influenza vaccine to children aged between 6 months and 5 years. Our study period was prior to this time. Relative to other ARI diagnoses, recorded influenza hospitalisation rates are low. Assessing the impact of the universal childhood vaccination program for influenza is likely to be challenging without linking national-level birth cohorts to infection surveillance and vaccination data. This has already been implemented in Scotland.[40] There is also renewed interest

For peer review only - http://bmjopen.bmj.com/site/about/guidelines.xhtml

in preventing morbidity due to RSV with vaccination.[41] Understanding the baseline hospitalisation rates for RSV-bronchiolitis and pneumonia prior to when vaccination is available is critical to aid in implementation and for its ongoing evaluation post implementation.

We conducted our analysis on near total population birth cohorts in each jurisdiction and thus our outcome measures have narrow confidence intervals and minimum selection bias. An additional strength of the population-based cohort design is standardisation of analysis protocols and the provision of large numbers allowing us to assess temporal trends and associations with less common infections. The hospital morbidity database systems used in all three jurisdictions have the same population coverage of all inpatient admissions and day surgeries further adding to the validity of our estimates. Although Western Australia is a state within Australia and we have made comparisons to country wide data for England and Scotland, the rate of cross-border hospitalisation from Western Australia to other Australian states is very low.[42]

However, our study does have some limitations. The socio-economic deprivation scores used were jurisdiction specific and included different items to represent disadvantage. In addition, area-level socio-economic deprivation was only measured at birth. Therefore, the observed association between area-level socio-economic deprivation and the rate of ARI admissions may be subject to increasing measurement error as the child's age increases. How socio-economic deprivation is associated with morbidity due to ARI at the primary care level is unknown but perhaps likely to aid in explaining disparities in socio-economic deprivation that we have seen here. While primary care data is more readily available in England and Scotland, limited data with adequate diagnostic information is available for population-based studies in Western Australia. As previously alluded to, there also may be differences in admission thresholds across the three jurisdictions that may explain

For peer review only - http://bmjopen.bmj.com/site/about/guidelines.xhtml

some higher admission rates across countries. A comparison of emergency department presentations in conjunction with hospitalisations for ARI could be useful here, although diagnostic information from emergency department data is limited[43] and no individual level data on emergency department visits exist in Scotland. Additionally, through our experience of linking routine laboratory data to hospital data in Western Australia, we are aware of unspecific ICD codes that are associated with detections of respiratory viral pathogens.[44] We did not include such ICD codes (e.g. viral infection of unspecified site “B34”) in this analysis. However we would not expect the potential exclusion of ARI hospitalisations to alter the direction of our results in terms of association with socio-economic deprivation.

CONCLUSIONS

Population-based administrative data from economically similar developed countries provides a powerful tool to conduct international comparative studies that can compare and contrast the epidemiology of, and healthcare responses to, respiratory infections. Western Australia experiences higher admissions in children for ARI and a greater disparity in rates according to level of socio-economic deprivation. Rates are overall slightly lower in England and Scotland but are increasing, particularly in England. These findings suggest that prevention programs focusing on disadvantaged populations in all three countries are likely to translate into real improvements in the burden of ARI in children. We are planning to use these administrative data to assess effectiveness of interventions (such as vaccination) and how this may affect disparities in ARI admissions rates according to socio-economic deprivation.

Acknowledgements

For peer review only

We would like to acknowledge the Western Australian Data Custodians, and particularly Alexandra Merchant and Mikhalina Dombrovskaya from the Western Australian Data Linkage Branch, for their assistance and support in collating the data for Western Australia. Source data from England can be accessed by researchers applying to the Health and Social Care Information Centre for England.
Copyright © 2017, Re-used with the permission of NHS Digital. All rights reserved.
This research benefits from and contributes to the NIHR Children and Families Policy Research Unit, but was not commissioned by the NIHR Policy Research Programme.

List of Abbreviations

ALRI: Acute lower respiratory infection; ARI: Acute respiratory infection; CI: Confidence interval; HES: Hospital Episode Statistics; ICD: International Classification of Diseases; IMD: Index of Multiple Deprivation; IRR: Incidence rate ratio; IRSAD: Index of Relative Disadvantage; NHS: National Health Service; RSV: Respiratory syncytial virus; SEIFA: Socio-Economic Index for Australia

Competing interests

The authors do not have any commercial or other association that might pose a conflict of interest.

Funding

This work was supported by a National Health and Medical Research Council Project Grant [1045668], a University of Western Australia Research Collaboration Award [to HCM] and a Wesfarmers Centre of Vaccines & Infectious Diseases Seed Grant [to HCM, CCB, PH]. CCB and HCM are supported by National Health and Medical Research Council Fellowships [1034254 to HCM and 1111596 to CCB]. PH was funded by a National Institute for Health Research postdoctoral

For peer review only - http://bmjopen.bmj.com/site/about/guidelines.xhtml

Fellowship, reference number PDF-2013–06–004. The views expressed in this publication are those of the author(s) and not necessarily those of the NHS, the National Institute for Health Research or the Department of Health. AZ's PhD studentship is funded by awards to establish the Farr Institute of Health Informatics Research, London, from the Medical Research Council, Arthritis Research UK, British Heart Foundation, Cancer Research UK, Chief Scientist Office, Economic and Social Research Council, Engineering and Physical Sciences Research Council, National Institute for Health Research, National Institute for Social Care and Health Research, and Wellcome Trust (grant MR/K006584/1). AZ is also supported by the Administrative Data Research Centre for England (funded by the Economic and Social Research Council). MV's PhD studentship is funded by the UBEL DTP of the Economic and Social Research Council. MV was also supported by the Policy Research Unit in the Health of Children, Young People and Families (reference 109/00017), which is funded by the Department of Health Policy Research Programme at UCL. This is an independent report commissioned and funded by the Department of Health. The views expressed are not necessarily those of the Department.

Ethics approval and consent to participate

Approval to use the Western Australian data was granted by the Western Australian Department of Health Human Research Ethics Committee, the Western Australian Aboriginal Health Ethics Committee and the Western Australian Data Linkage Branch. We have a data sharing agreement with National Health Service (NHS) Digital to use a de-identified extract of Hospital Episode Statistics for research into children's use of secondary care services; therefore, we did not require ethical approval to use English datasets. For Scotland, approvals were obtained from the Public Benefit and Privacy Panel for Health and Social Care, reference number 1516-0405.

Consent for publication

For peer review only - http://bmjopen.bmj.com/site/about/guidelines.xhtml

Not applicable.

Data Sharing Statement

We cannot share the individual-level data used for this study under our agreements with the data providers. The datasets analysed during the current study can be applied for from the Western Australian Data Linkage System (Western Australia; http://www.datalinkage-wa.org.au/), NHS Digital; (England; http://content.digital.nhs.uk) and the electronic Data Research and Innovation Service (Scotland; http://www.isdscotland.org/Products-and-Services/eDRIS/). Derived data from these datasets for each jurisdiction are within the paper. No additional data are available.

Authors' contributions

HCM, CCB and PH conceived the study design. PF assisted with data cleaning and coding in Western Australia, AZ and MV assisted with data extraction for England and Scotland. Statistical analysis was conducted by HCM (Western Australia) and PH (England and Scotland) with expert advice from NdK with critical revisions for intellectual content from CCB and RG. HCM drafted the first manuscript with PH. All authors read and approved the final manuscript.

REFERENCES

For peer review only - http://bmjopen.bmj.com/site/about/guidelines.xhtml

1. Nair H, Simoes EA, Rudan I*, et al.* Global and regional burden of hospital admissions for severe acute lower respiratory infections in young children in 2010: a systematic analysis. *Lancet*. 2013;**381**:1380-1390.

2. Shi T, McAllister DA, O'Brien KL*, et al.* Global, regional, and national disease burden estimates of acute lower respiratory infections due to respiratory syncytial virus in young children in 2015: a systematic review and modelling study. *Lancet*. 2017;**390**:946-958.

3. Gill PJ, Goldacre MJ, Mant D*, et al.* Increase in emergency admissions to hospital for children aged under 15 in England, 1999–2010: national database analysis. *Arch Dis Child*. 2013.

4. Harron K, Gilbert R, Cromwell D*, et al.* International comparison of emergency hospital use for infants: data linkage cohort study in Canada and England. *BMJ Quality & Safety*. 2017.

5. Moore H, Burgner D, Carville K*, et al.* Diverging trends for lower respiratory infections in non-Aboriginal and Aboriginal children. *J Paediatr Child Health*. 2007;**43**:451-457.

6. Moore HC, Lehmann D, de Klerk N*, et al.* Reduction in disparity for pneumonia hospitalisations between Australian Indigenous and non-Indigenous children. *Journal of epidemiology and community health*. 2012;**66**:489-494.

7. Anderson AJ, Snelling TL, Moore HC*, et al.* Advances in Vaccines to Prevent Viral Respiratory Illnesses in Children. *Paediatr Drugs*. 2017;**19**:523-531.

8. Cakmak S, Hebbern C, Cakmak JD*, et al.* The modifying effect of socioeconomic status on the relationship between traffic, air pollution and respiratory health in elementary schoolchildren. *J Environ Manage*. 2016;**177**:1-8.

9. Moore HC, de Klerk N, Richmond P*, et al.* A retrospective population-based cohort study identifying target areas for prevention of acute lower respiratory infections in children. *BMC Public Health*. 2010;**10**:757.

10. MacIntyre EA, Gehring U, Mölter A*, et al.* Air pollution and respiratory infections during early childhood: an analysis of 10 European birth cohorts within the ESCAPE Project. *Environ Health Perspect*. 2014;**122**:107.

For peer review only

For peer review only - http://bmjopen.bmj.com/site/about/guidelines.xhtml

11. Smith KR, Samet JM, Romieu I, *et al.* Indoor air pollution in developing countries and acute lower respiratory infections in children. *Thorax*. 2000;**55**:518-532.

12. Health AGDo. The Australian Immunisation Handbook 10th edition (updated February 2017): Australian Government; 2017.

13. Public Health England. Quarterly vaccination coverage statistics for children aged up to five years in the UK (COVER programme): July to September 20182018 14 Dec 2018.

14. ABS. 3101.0 - Australian Demographic Statistics, Jun 2014. Australian Bureau of Statistics; 2015; Available from: http://www.abs.gov.au/AUSSTATS/abs@.nsf/Lookup/3101.0Main+Features1Jun%202014?OpenDocument.

15. HealthInfoNet. What details do we know about the Indigenous population? : Australian Indigenous HealthInfoNet 2015; Available from: http://www.healthinfonet.ecu.edu.au/health-facts/health-faqs/aboriginal-population.

16. Holman CD, Bass AJ, Rosman DL, *et al.* A decade of data linkage in Western Australia: strategic design, applications and benefits of the WA data linkage system. *Aust Health Rev*. 2008;**32**:766-777.

17. Kelman CW, Bass AJ, Holman C. Research use of linked health data—a best practice protocol. *Aust N Z J Public Health*. 2002;**26**:251-255.

18. Christenen D, Davis G, Draper G, *et al.* Evidence for the use of an algorithm in resolving inconsistent and missing Indigenous status in administrative data collections. *Australian Journal of Social Issues*. 2014;**49**:423-443.

19. Office for National Statistics. 2011 Census: Population Estimates for the United Kingdom, March 2011. 2012 [6 February 2018]; Available from: https://www.ons.gov.uk/peoplepopulationandcommunity/populationandmigration/populationestimates/bulletins/2011censuspopulationestimatesfortheunitedkingdom/2012-12-17.

For peer review only

For peer review only - http://bmjopen.bmj.com/site/about/guidelines.xhtml

20. Herbert A, Wijlaars L, Zylbersztejn A*, et al.* Data Resource Profile: Hospital Episode Statistics Admitted Patient Care (HES APC). *Int J Epidemiol*. 2017;**46**:1093-1093i.

21. NHS Digital. A Guide to Linked Mortality Data from Hospital Episode Statistics and the Office for National Statistics. 2015 [6 February 2018]; Available from: http://content.digital.nhs.uk/media/11668/HES-ONS-Mortality-Data-Guide/pdf/mortality_guide.pdf.

22. Administrative Data Liaison Service. Birth Registrations - National Records of Scotland. 2016 [4 November 2016]; Available from: http://www.adls.ac.uk/national-records-of-scotland/birth-registrations/?detail.

23. Administrative Data Liaison Service. SMR02 – Maternity Inpatient and Day Case dataset. 2016 [4 November 2016]; Available from: http://www.adls.ac.uk/nhs-scotland/maternity-inpatient-and-day-case-smr02/?detail.

24. Harron K, Gilbert R, Cromwell D*, et al.* Linking Data for Mothers and Babies in De-Identified Electronic Health Data. *PLoS ONE*. 2016;**11**:e0164667.

25. Roberts RF, Innes KC, Walker SM. Introducing ICD-10-AM in Australian hospitals. *The Medical Journal of Australia*. 1998;**169**:S32-35.

26. Pink B. An Introduction to Socio-Economic Indexes for Areas (SEIFA) - Information paper: Australian Bureau of Statistics, Commonwealth of Australia 2008.

27. Smith T, Noble M, Noble S*, et al.* The English Indices of Deprivation 2015: Technical Report: Department for Communities and Local Government 2015.

28. Brown D, Allik M, Dundas R*, et al.* Carstairs Scores for Scottish Postcode Sectors, Datazones & Output Areas from the 2011 Census: University of Glasgow 2014.

29. Gonzalez-Izquierdo A, Cortina-Borja M, Woodman J*, et al.* Maltreatment or violence-related injury in children and adolescents admitted to the NHS: comparison of trends in England and Scotland between 2005 and 2011. *BMJ Open*. 2014;**4**:e004474.

30. StataCorp. Stata Statistical Software. Release 14. College Station, TX.: StataCorp LP.; 2015.

For peer review only - http://bmjopen.bmj.com/site/about/guidelines.xhtml

31. Kivistö JE, Protudjer JLP, Karjalainen J*, et al.* Trends in paediatric asthma hospitalisations – differences between neighbouring countries. *Thorax*. 2017.

32. Department of Health. Emergecny admissions to hospital: managing the demand: National Audit Office 2013.

33. Hawker JI, Olowokure B, Sufi F*, et al.* Social deprivation and hospital admission for respiratory infection:: an ecological study. *Respir Med*. 2003;**97**:1219-1224.

34. Zubrick SR, Lawrence D, Silburn S*, et al.* The Western Australian Aboriginal Child Health Survey: The Health of Aboriginal Children and Young People 1ed. Perth, Western Australia: Telethon Institute for Child Health Research 2004.

35. Holman RC, Hennessy TW, Haberling DL*, et al.* Increasing trend in the rate of infectious disease hospitalisations among Alaska Native people. *Int J Circumpolar Health*. 2013;**72**:20994.

36. O'Grady K-Ann F, Lee Katherine J, Carlin John B*, et al.* Increased risk of hospitalization for acute lower respiratory tract infection among Australian Indigenous infants 5-23 months of age following pneumococcal vaccination: A cohort study. *Clin Infect Dis*. 2010;**50**:970-978.

37. Jardine A, Menzies RI, McIntyre PB. Reduction in Hospitalizations for Pneumonia Associated With the Introduction of a Pneumococcal Conjugate Vaccination Schedule Without a Booster Dose in Australia. *Pediatr Infect Dis J*. 2010;**29**:000-000.

38. Moore HC, Lim FJ, Fathima P*, et al.* Exploring RSV epidemiology in Australian chidlren: estimates of the population burden and effectiveness of a maternal vaccine. **RSV Vaccines for the World**; 29 Nov - 1 Dec; Malaga, Spain2017.

39. Thomas SL, Wakerman J, Humphreys JS. Ensuring equity of access to primary health care in rural and remote Australia - what core services should be locally available? *Int J Equity Health*. 2015;**14**:111.

40. Hardelid P, Verfuerden M, McMenamin J*, et al.* Risk factors for admission to hospital with laboratory-confirmed influenza in young children: birth cohort study. *Eur Respir J*. 2017;**50**:1700489.

For peer review only - http://bmjopen.bmj.com/site/about/guidelines.xhtml

41. World Health Organization. RSV Vaccine Research and Develoment Technology Roadmap2017.

42. Spilsbury K, Rosman D, Alan J*, et al.* Cross border hospital use: analysis using data linkage across four Australian states. *Med J Aust*. 2015;**202**:582-586.

43. Moore HC, de Klerk N, Jacoby P*, et al.* Can linked emergency department data help assess the out-of-hospital burden of acute lower respiratory infections? A population-based cohort study. *BMC Public Health*. 2012;**12**:703.

44. Lim F, Blyth CC, Fathima P*, et al.* Record linkage study of the pathogen-specific burden of respiratory viruses in children. *Influenza & Other Respiratory Viruses*. 2017;**11**:502-510.

For peer review only

For peer review only - http://bmjopen.bmj.com/site/about/guidelines.xhtml

Figure legends

Figure 1: Hospitalisation rates for ARI in Western Australia, England and Scotland by level of socio-economic deprivation for A) infants (<1 year) and B) young children (1-4 years). Those in the <10% level represent the most deprived and those ≥ 90% represent those least deprived.

Figure 2: Hospitalisation rates by year of admission for infants (<1 year) and children (1-4 years) in Western Australia, England and Scotland for ARI, bronchiolitis, pneumonia and unspecified ALRI.

For peer review only

Table 1: Number of admissions and hospitalisation rate for ARI by diagnostic category by principal diagnosis in infants aged <1 year and children aged 1-4 years in Western Australia, England and Scotland

Diagnosis	Western Australia				England				Scotland			
	n	(%)	Rate[a]	(95% CI)	n	(%)	Rate[a]	(95% CI)	n	(%)	Rate[a]	(95% CI)
**<1 year[b]**												
Whooping cough	220	(1.6)	0.7	(0.6, 0.8)	2395	(1.1)	0.5	(0.4, 0.5)	372	(1.4)	0.6	(0.5, 0.6)
Pneumonia	1278	(9.4)	4.1	(3.9, 4.4)	15592	(7.2)	2.9	(2.9, 3.0)	1245	(4.8)	1.9	(1.8, 2.1)
Bronchiolitis	10,652	(78.7)	34.4	(33.8, 35.1)	171805	(78.8)	32.2	(32.1, 32.4)	22021	(84.4)	34.3	(33.9, 34.8)
Influenza	407	(3.0)	1.3	(1.2, 1.4)	1627	(0.7)	0.3	(0.3, 0.3)	426	(1.6)	0.7	(0.6, 0.7)
Unspecified ALRI	809	(6.0)	2.6	(2.4, 2.8)	24563	(11.3)	4.6	(4.5, 4.7)	1797	(6.9)	2.8	(2.7, 2.9)
Bronchitis	169	(1.2)	0.5	(0.5, 0.6)	2003	(0.9)	0.4	(0.4, 0.4)	242	(0.9)	0.4	(0.3, 0.4)
All ARI	13,535	(100.0)	43.7	(43.0, 44.5)	217985	(100.0)	40.1	(40.7, 41.1)	26103	(100.0)	40.7	(40.2, 41.2)
**1-4 years[c]**												
Whooping cough	33	(0.4)	0.04	(0.03, 0.06)	95	(0.1)	0.008	(0.007, 0.01)	23	(0.2)	0.01	(0.01,0.02)
Pneumonia	3031	(41.6)	3.7	(3.6, 3.9)	29741	(33.2)	2.5	(2.5, 2.6)	3411	(26.9)	2.1	(2.0, 2.1)
Bronchiolitis	1893	(26.0)	2.3	(2.2, 2.4)	8283	(9.2)	0.7	(0.7, 0.7)	3141	(24.7)	1.9	(1.8, 2.0)
Influenza	366	(5.0)	0.4	(0.4, 0.5)	1714	(1.9)	0.2	(0.1, 0.2)	392	(3.1)	0.2	(0.2, 0.3)
Unspecified ALRI	1767	(24.3)	2.2	(2.1, 2.3)	48910	(54.6)	4.2	(4.1, 4.2)	5570	(43.9)	3.3	(3.3, 3.4)
Bronchitis	195	(2.7)	0.2	(0.2, 0.3)	859	(1.0)	0.1	(0.1, 0.1)	161	(1.3)	0.1	(0.1, 0.1)
All ALRI	7285	(100.0)	9.0	(8.8, 9.2)	89602	(100.0)	7.6	(7.6, 7.7)	12698	(100.0)	7.6	(7.5, 7.8)

[a]Rate is per 1000/child-years

[b]2001-2012 for Western Australia and Scotland; 2004-2012 for England

For peer review only - http://bmjopen.bmj.com/site/about/guidelines.xhtml

[c]2005-2012 for Western Australia and Scotland; 2008-2012 for England

For peer review only

For peer review only - http://bmjopen.bmj.com/site/about/guidelines.xhtml

Table 2: Risk of hospitalisation for bronchiolitis, pneumonia, unspecified ALRI and overall ARI from log-linear modelling in infants aged <1 year in Western Australia, England and Scotland

Exposure	Western Australia		England		Scotland	
	IRR	(95% CI)	IRR	(95% CI)	IRR	(95% CI)
**Bronchiolitis**						
Year[a]	0.99	(0.98, 1.00)	1.05	(1.04, 1.07)	1.04	(1.03, 1.05)
Male	*Reference*		*Reference*		*Reference*	
Female	0.68	(0.64, 0.72)	0.68	(0.63, 0.74)	0.70	(0.64, 0.77)
Deprivation <10%	3.34	(3.02, 3.71)	1.94	(1.73, 2.19)	1.28	(1.16, 1.42)
Deprivation 10-49%	2.04	(1.75, 2.37)	1.48	(1.07, 2.06)	1.29	(0.89, 1.87)
Deprivation 50-89%	1.36	(1.19, 1.55)	1.18	(0.95, 1.45)	1.09	(0.85, 1.40)
Deprivation ≥90%	*Reference*		*Reference*		*Reference*	
Non-ARI admissions	1.00	(1.00, 1.00)	1.00	1.00, 1.00)	1.00	(1.00, 1.00)
**Pneumonia**						
Year[a]	0.94	(0.93, 0.96)	0.98	(0.97, 0.99)	0.97	(0.94, 0.99)
Male	*Reference*		*Reference*		*Reference*	
Female	0.75	(0.67, 0.84)	0.76	(0.70, 0.82)	0.80	(0.67, 0.97)
Deprivation 0-10%	6.91	(5.59, 8.56)	1.47	(1.30, 1.66)	1.09	(0.88, 1.37)
Deprivation 10-49%	3.26	(2.49, 4.28)	0.90	(0.65, 1.25)	0.74	(0.39, 1.43)
Deprivation 50-89%	1.66	(1.29, 2.13)	0.86	(0.70, 1.07)	0.80	(0.51, 1.25)
Deprivation >90%	*Reference*		*Reference*		*Reference*	
Non-ARI admissions	1.00	(1.00, 1.00)	1.00	(1.00, 1.00)	1.00	(1.00, 1.00)
**Unspecified ALRI**						
Year[a]	0.95	(0.93, 0.97)	1.06	(1.05, 1.07)	1.02	(1.00, 1.04)
Male	*Reference*		*Reference*		*Reference*	
Female	0.62	(0.54, 0.71)	0.65	(0.61, 068)	0.73	(0.62, 0.85)
Deprivation <10%	8.90	(6.69, 11.83)	1.81	(1.66, 1.98)	0.93	(0.78, 1.12)
Deprivation 10-49%	4.18	(2.93, 5.96)	1.34	(1.06, 1.70)	0.84	(0.48, 1.48)
Deprivation 50-89%	1.96	(1.40, 2.73)	1.11	(0.95, 1.30)	0.85	(0.58, 1.26)
Deprivation >90%	*Reference*		*Reference*		*Reference*	
Non-ARI admissions	1.00	(1.00, 1.00)	1.00	(1.00, 1.00)	1.00	(1.00, 1.00)
**Total ARI**						
Year[a]	0.99	(0.98, 1.00)	1.05	(1.04,1.06)	1.03	(1.02, 1.04)
Male	*Reference*		*Reference*		*Reference*	

For peer review only - http://bmjopen.bmj.com/site/about/guidelines.xhtml

Female	0.68	(0.65, 0.72)	0.69	(0.64, 0.73)	0.71	(0.65, 0.77)
Deprivation 0-10%	3.85	(3.50, 4.21)	1.87	(1.70, 2.06)	1.25	(1.14, 1.37)
Deprivation 10-49%	2.22	(1.95, 2.54)	1.41	(1.07, 1.85)	1.25	(0.88, 1.77)
Deprivation 50-89%	1.42	(1.26, 1.60)	1.14	(0.96, 1.36)	1.08	(0.85, 1.36)
Deprivation >90%	*Reference*		*Reference*		*Reference*	
Non-ARI admissions	1.00	(1.00, 1.00)	1.00	(1.00, 1.00)	1.00	(1.00, 1.00)

IRR, Incidence Rate Ratio

[a]Year included as a linear term

For peer review only

For peer review only - http://bmjopen.bmj.com/site/about/guidelines.xhtml

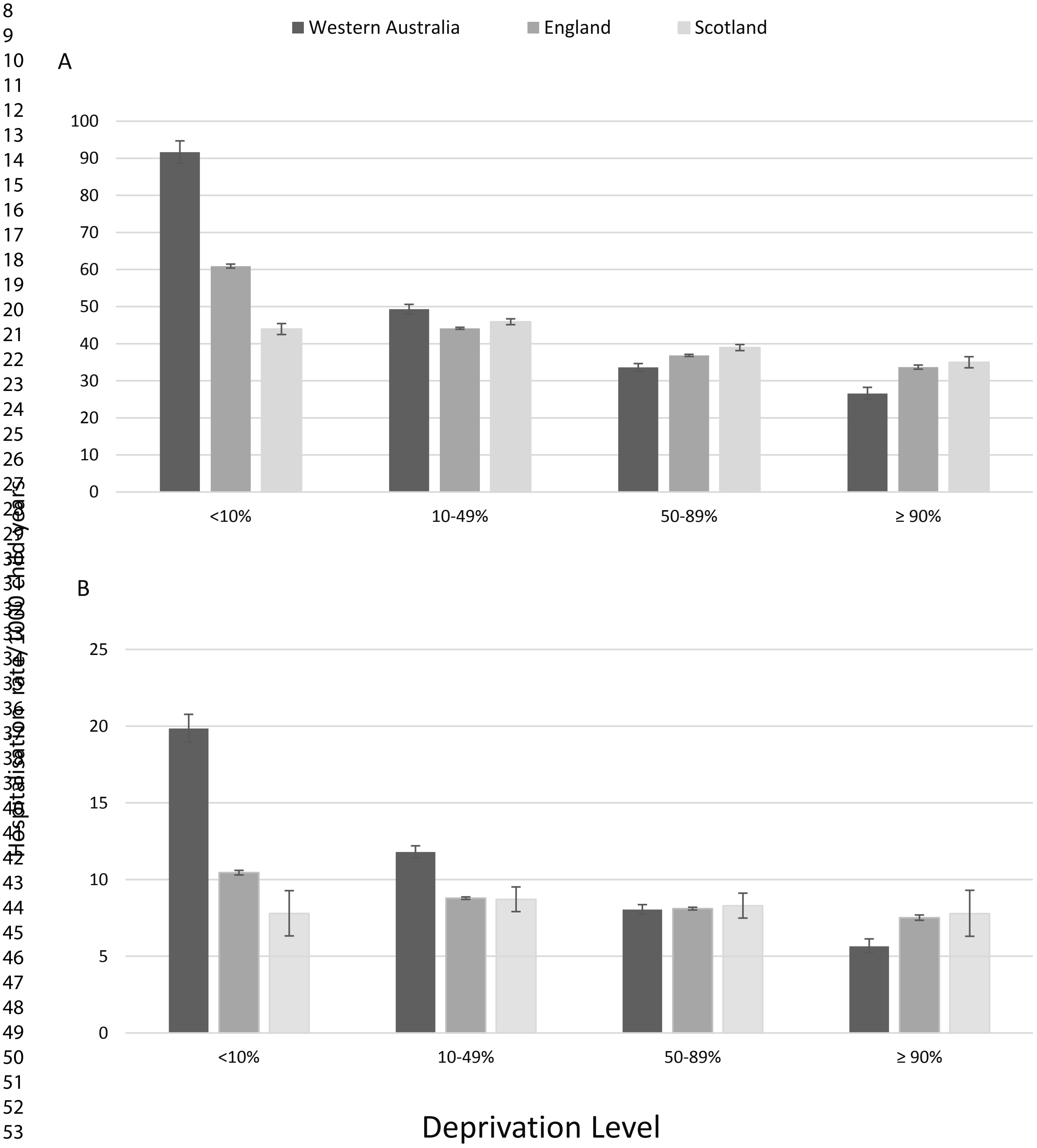
Western Australia
England
Scotland
A
100
90
80
70
60
50
40
30
20
10
0
<10%
10-49%
50-89%
≥ 90%
B
25
20
15
10
5
0
<10%
10-49%
50-89%
≥ 90%
Hospitalisation rate/1000 child years
Deprivation Level

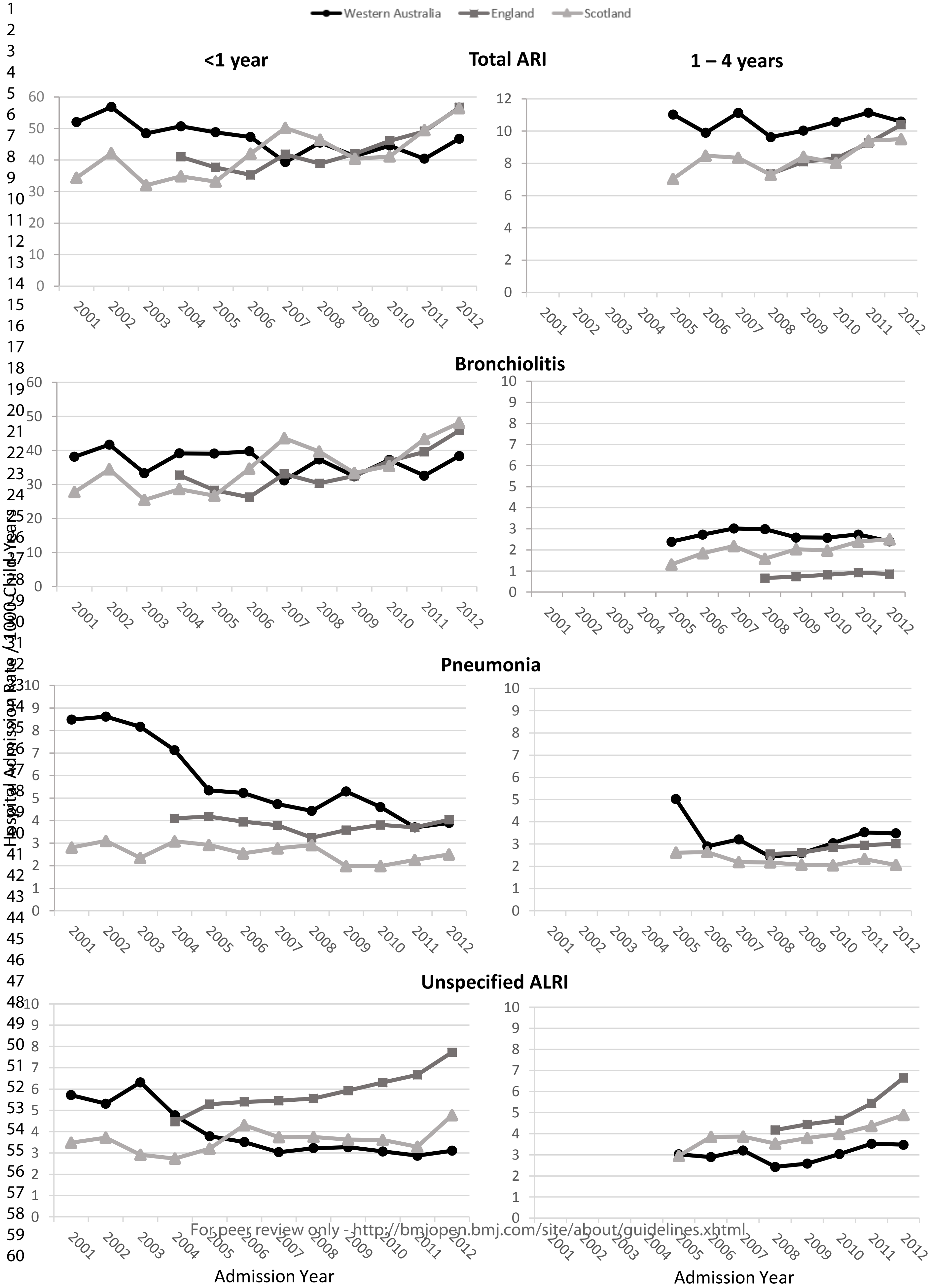

For peer review only - http://bmjopen.bmj.com/site/about/guidelines.xhtml

Supplementary Table 1: Cohort characteristics by jurisdiction

Characteristic		Western Australia		England		Scotland	
		n	(%)	n	(%)	n	(%)
Sex[a]	Male	173,081	(51.2)	3,044,931	(51.3)	359,159	(51.3)
	Female	164,828	(48.8)	2,894,078	(48.7)	340,430	(48.7)
Year of birth	2000	22,551	(6.7)	-		51,479	(7.4)
	2001	22,461	(6.7)	-		50,295	(7.2)
	2002	22,412	(6.7)	-		49,425	(7.1)
	2003	22,345	(6.6)	535,724	(9.0)	50,450	(7.2)
	2004	23,361	(6.9)	551,939	(9.3)	52,417	(7.5)
	2005	24,776	(7.3)	560,894	(9.4)	52,415	(7.5)
	2006	26,627	(7.9)	579,220	(9.8)	53,830	(7.7)
	2007	26,943	(8.0)	586,526	(9.9)	55,765	(8.0)
	2008	28,216	(8.4)	614,471	(10.4)	57,481	(8.2)
	2009	28,588	(8.5)	611,427	(10.3)	57,007	(8.2)
	2010	28,565	(8.5)	629,778	(10.7)	56,586	(8.1)
	2011	29,535	(8.7)	632,306	(10.7)	56,510	(8.1)
	2012	31,529	(9.3)	636,724	(10.7)	55,929	(8.0)
Deprivation[b]	<10% (most deprived)	40,936	(12.1)	885,891	(14.9)	85,987	(12.3)
	10-49%	122,309	(36.2)	2,635,562	(44.4)	295,372	(42.2)
	50-89%	131,232	(38.8)	1,957,789	(33.0)	252,407	(36.1)
	≥90% (least deprived)	43,432	(12.9)	459,767	(7.6)	65,823	(9.4)
**TOTAL**		**337,909**	**(100.0)**	**5,939,009**	**(100.0)**	**699,589**	**(100.0)**

[a] Sex was missing was 7205 children (England) with no missing data from Western Australia or Scotland.

[b] Deprivation scores were missing for 18,920 children (Western Australia), 146,588 (England) and 827 (Scotland).

For peer review only

Supplementary Table 2: Hospitalisation rate for ARI by diagnostic category by primary diagnosis only (PDx) and primary diagnosis plus all additional diagnoses (Any Dx) in infants aged <1 year and children aged 1-4 years in Western Australia, England and Scotland

	Western Australia			England			Scotland		
	Rate[a]		IRR (95% CI)	Rate[a]		IRR (95% CI)	Rate[a]		IRR (95% CI)
**ARI diagnosis**	**Any Dx**	**PDx**		**Any Dx**	**PDx**		**Any Dx**	**PDx**	
**<1 year[b]**									
Whooping cough	0.8	0.7	1.2 (1.0, 1.4)	0.5	0.5	1.1 (1.0, 1.2)	0.6	0.6	1.1 (0.9, 1.2)
Pneumonia	5.6	4.1	1.4 (1.3, 1.5)	3.8	2.9	1.3 (1.3, 1.3)	2.6	1.9	1.3 (1.2, 1.4)
Bronchiolitis	36.6	34.4	1.1 (1.0, 1.1)	34.2	32.2	1.1 (1.1, 1.1)	35.4	34.3	1.0 (1.0, 1.1)
Influenza	1.6	1.3	1.3 (1.1, 1.4)	0.4	0.3	1.4 (1.3, 1.5)	0.9	0.7	1.3 (1.2, 1.5)
Unspecified ARI	3.9	2.6	1.5 (1.4, 1.6)	5.9	4.6	1.3 (1.3, 1.3)	3.6	2.8	1.3 (1.2, 1.4)
Bronchitis	0.7	0.5	1.3 (1.1, 1.6)	0.5	0.4	1.2 (1.1, 1.3)	0.5	0.4	1.2 (1.1, 1.5)
Total ARI	46.4	43.7	1.1 (1.0, 1.1)	43.4	40.1	1.1 (1.1, 1.1)	42.1	40.7	1.0 (1.0, 1.1)
**1-4 years[c]**									
Whooping cough	0.05	0.04	1.1 (0.7, 1.9)	0.009	0.008	1.1 (0.9, 1.5)	0.02	0.01	1.1 (0.7, 2.0)
Pneumonia	4.3	3.7	1.2 (1.1, 1.2)	2.8	2.5	1.1 (1.1, 1.1)	2.3	2.1	1.1 (1.1, 1.1)
Bronchiolitis	2.7	2.3	1.2 (1.1, 1.2)	0.8	0.7	1.1 (1.1, 1.2)	2.0	1.9	1.1 (1.0, 1.1)
Influenza	0.6	0.4	1.3 (1.1, 1.4)	0.2	0.2	1.3 (1.2, 1.4)	0.3	0.2	1.2 (1.1, 1.4)
Unspecified ARI	3.0	2.2	1.4 (1.3, 1.5)	5.1	4.2	1.2 (1.2, 1.2)	3.9	3.3	1.2 (1.1, 1.2)
Bronchitis	0.3	0.2	1.4 (1.2, 1.7)	0.1	0.1	1.2 (1.1, 1.3)	0.1	0.1	1.3 (0.0, 1.6)
Total ARI	10.5	9.0	1.2 (1.1, 1.2)	8.7	7.6	1.1 (1.1, 1.1)	8.4	7.6	1.1 (0.0, 1.1)

IRR, incidence rate ratio

For peer review only

[a]Rate is per 1000/child-years

[b]2001-2012 for Western Australia and Scotland; 2004-2012 for England

[c]2005-2012 for Western Australia and Scotland; 2008-2012 for England

For peer review only

STROBE Statement—Checklist of items that should be included in reports of ***cohort studies***

	Item No	Recommendation	Page No
**Title and abstract**	1	(*a*) Indicate the study's design with a commonly used term in the title or the abstract	1
		(*b*) Provide in the abstract an informative and balanced summary of what was done and what was found	2
**Introduction**			
Background/rationale	2	Explain the scientific background and rationale for the investigation being reported	4
Objectives	3	State specific objectives, including any prespecified hypotheses	5
**Methods**			
Study design	4	Present key elements of study design early in the paper	5
Setting	5	Describe the setting, locations, and relevant dates, including periods of recruitment, exposure, follow-up, and data collection	5-6
Participants	6	(*a*) Give the eligibility criteria, and the sources and methods of selection of participants. Describe methods of follow-up	5-6
		(*b*) For matched studies, give matching criteria and number of exposed and unexposed	
Variables	7	Clearly define all outcomes, exposures, predictors, potential confounders, and effect modifiers. Give diagnostic criteria, if applicable	6-7
Data sources/ measurement	8*	For each variable of interest, give sources of data and details of methods of assessment (measurement). Describe comparability of assessment methods if there is more than one group	7
Bias	9	Describe any efforts to address potential sources of bias	8
Study size	10	Explain how the study size was arrived at	6
Quantitative variables	11	Explain how quantitative variables were handled in the analyses. If applicable, describe which groupings were chosen and why	7-8
Statistical methods	12	(*a*) Describe all statistical methods, including those used to control for confounding	8
		(*b*) Describe any methods used to examine subgroups and interactions	8
		(*c*) Explain how missing data were addressed	8, Additional File 1
		(*d*) If applicable, explain how loss to follow-up was addressed	
		(*e*) Describe any sensitivity analyses	
**Results**			
Participants	13*	(a) Report numbers of individuals at each stage of study—eg numbers potentially eligible, examined for eligibility, confirmed eligible, included in the study, completing follow-up, and analysed	9
		(b) Give reasons for non-participation at each stage	
		(c) Consider use of a flow diagram	
Descriptive data	14*	(a) Give characteristics of study participants (eg demographic, clinical, social) and information on exposures and potential confounders	Additional File 1
		(b) Indicate number of participants with missing data for each variable of interest	Additional File 1
		(c) Summarise follow-up time (eg, average and total amount)	9
Outcome data	15*	Report numbers of outcome events or summary measures over time	9

For peer review only - http://bmjopen.bmj.com/site/about/guidelines.xhtml

Main results	16	(*a*) Give unadjusted estimates and, if applicable, confounder-adjusted estimates and their precision (eg, 95% confidence interval). Make clear which confounders were adjusted for and why they were included<br>(*b*) Report category boundaries when continuous variables were categorized<br>(*c*) If relevant, consider translating estimates of relative risk into absolute risk for a meaningful time period	23-26
Other analyses	17	Report other analyses done—eg analyses of subgroups and interactions, and sensitivity analyses	13
**Discussion**			
Key results	18	Summarise key results with reference to study objectives	11
Limitations	19	Discuss limitations of the study, taking into account sources of potential bias or imprecision. Discuss both direction and magnitude of any potential bias	14
Interpretation	20	Give a cautious overall interpretation of results considering objectives, limitations, multiplicity of analyses, results from similar studies, and other relevant evidence	13-14
Generalisability	21	Discuss the generalisability (external validity) of the study results	14-15
**Other information**			
Funding	22	Give the source of funding and the role of the funders for the present study and, if applicable, for the original study on which the present article is based	16

*Give information separately for exposed and unexposed groups.

Note: An Explanation and Elaboration article discusses each checklist item and gives methodological background and published examples of transparent reporting. The STROBE checklist is best used in conjunction with this article (freely available on the Web sites of PLoS Medicine at http://www.plosmedicine.org/, Annals of Internal Medicine at http://www.annals.org/, and Epidemiology at http://www.epidem.com/). Information on the STROBE Initiative is available at http://www.strobe-statement.org.

For peer review only

For peer review only - http://bmjopen.bmj.com/site/about/guidelines.xhtml
